# Structural and phylogenetic analyses of umbravirus and umbra-like virus genomes suggest evolution of capsid-like proteins from 30K movement proteins

**DOI:** 10.1128/jvi.02209-25

**Published:** 2026-03-19

**Authors:** Anne E. Simon, Jason M. Needham, Anna Mikkelsen, Osama Atallah

**Affiliations:** 1 Department of Cell Biology and Molecular Genetics, University of Maryland College Park 1068, College Park, Maryland, USA; Tsinghua University, Beijing, China

**Keywords:** cap-independent translation enhancer, ISS 3ʹCITEs, -1 ribosomal frameshifting, RNA translation structures, virus evolution, virus movement proteins

## Abstract

Plant umbraviruses (UVs) encode, in embedded open reading frames, an unstructured movement protein (MP) (ORF3) required for vascular long-distance movement and a structured 30K-type MP (ORF4) for cell-to-cell movement. UVs do not encode capsid proteins and require helper viruses for vector acquisition. Umbra-like viruses (ULVs) comprise two groups whose RNA-dependent RNA polymerases share significant similarity with UVs and whose non-replicase ORFs, if any, are either single, embedded, or separate. Group 2 ULVs, which lack encoded MPs, use a host-encoded MP and do not require a helper virus, presumably due to encoding a capsid-like protein. Group 1 ULVs are more closely related to UVs and have been identified in Ageratum, Arborvitae, *Paspalum paspaloides*, switchgrass, and grapevine. Structural and phylogenetic analyses of Group 1 ULVs suggest that ULVs evolved from UVs following the loss of ORF3, duplication of 30K MP ORF 4, recombination to remove an intergenic region, and evolution of the paralog ORF4s to encode a capsid-like protein and a protein of unknown function. Neither duplicated ORF in Group 1 grapevine GULV4 was required for systemic infection of *Nicotiana benthamiana*. Structural comparisons of 17 UV and 8 Group 1 ULV frameshifting sites revealed strong conservation, including two previously unknown hairpins and pseudoknots that were absent in Group 2 ULVs. Group 1 ULVs also contain I-shaped structure (ISS) 3ʹ cap-independent translation enhancers with novel conformations not found in UVs or Group 2 ULVs. These and other findings comprise a baseline of knowledge that will inform further experimental examination of these unique viruses.

**IMPORTANCE:**

The key defining feature of plant viruses is their encoding of movement proteins (MPs), with most from a superfamily of MPs known as 30K. It has been proposed that 30K MPs evolved from capsid proteins, as both contain similar “jelly roll” domains. Umbraviruses encode two MPs in overlapping ORFs, with ORF4 encoding a 30K-type MP. Umbraviruses and umbra-like viruses (ULVs) have related replication proteins (ORFs 1 and 2) but differ in their non-replication ORFs. Recently, Group 2 ULVs were found to encode a capsid-like protein and no MPs, relying instead on host proteins for movement. Current investigation of the sequences and structures of Group 1 ULVs, which contain one or two ORFs of unknown function, revealed that ULVs likely evolved from umbraviruses following duplication of the 30K MP ORF and neofunctionalizations to a jelly roll-containing capsid-like protein and a second non-MP, suggesting that capsid proteins can also evolve from MPs.

## INTRODUCTION

**Fig 1:**
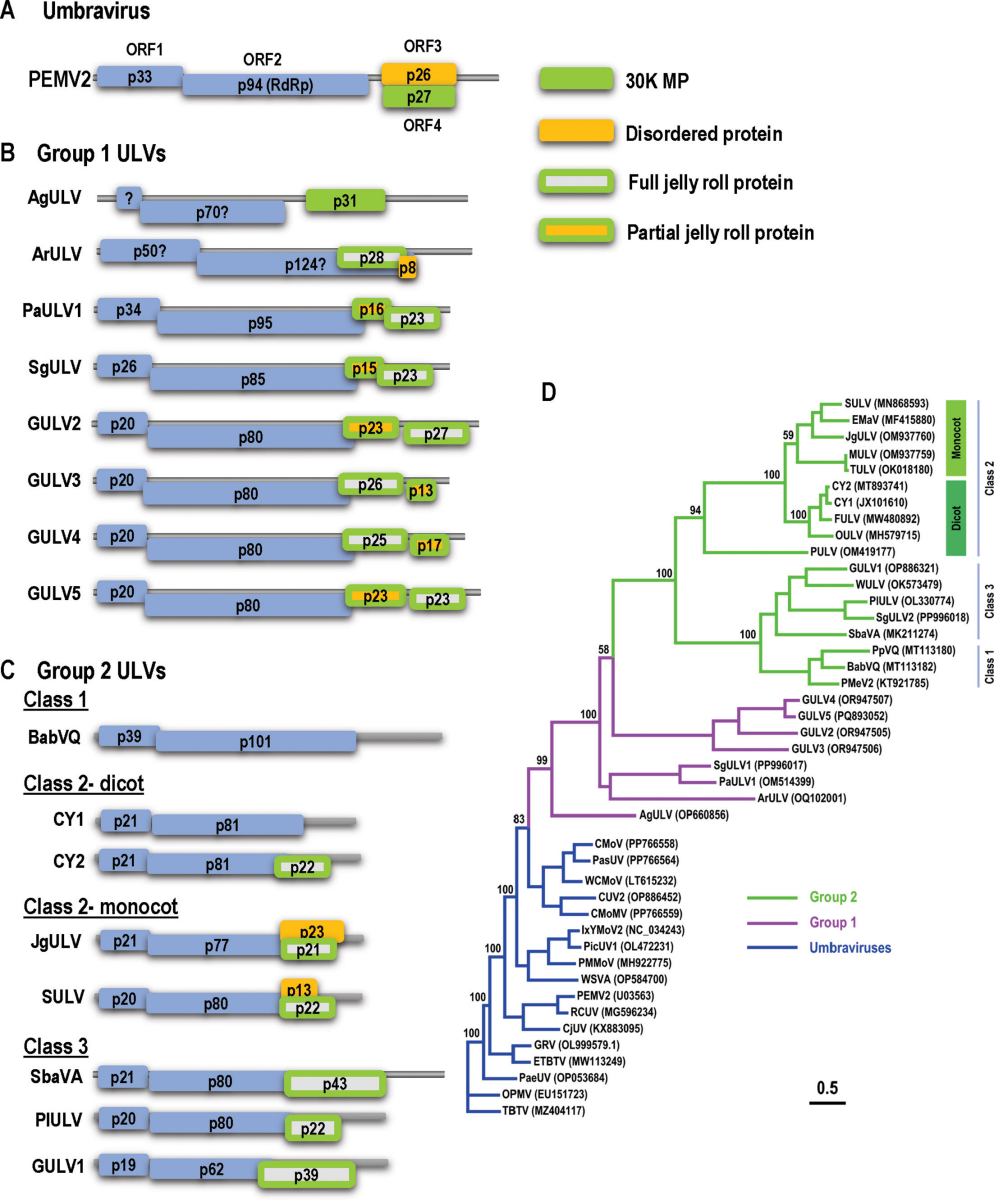
Genome organization and phylogenetic analysis of UVs and ULVs. (**A**) Gene organization of UV PEMV2. The two 5ʹ proximal ORFs encode replication proteins, including the RdRp, which requires a −1 frameshifting event near the ORF1 stop codon. ORF3 encodes a disordered, long-distance MP, and ORF4 encodes a 30K cell-to-cell MP. (**B**) Group 1 ULVs share similar RdRp with UVs but different downstream ORFs, which are color-coded according to the predicted structure of their encoded proteins (see Fig. 3). Question marks denote that annotated protein molecular masses are likely incorrect (see text). (**C**) Representative members of Group 2 ULVs. (**D**) Maximum-likelihood phylogenetic tree based on RdRp amino acid sequences out of 1,000 replicates. Bootstrap support values for the major clade nodes are indicated. Accession numbers are shown. Virus abbreviations are defined in Table S1. Scale bar represents the number of amino acid substitutions per site. The alignment used to generate this phylogenetic tree can be found in Data set S1.

Umbraviruses (UVs) are unusual members of the *Tombusviridae* due to their lack of encoded capsid proteins and requirement for co-infection with a helper virus from the luteovirus, enamovirus, or polerovirus genera (1). The 17 members of the umbravirus genus have single component, plus-sense RNA genomes ranging in size from 4.1 to 4.5 kb and contain four open reading frames (ORFs) (Fig. 1A). ORF1 encodes a mainly unstructured protein that, in related tombusviruses, is important for assembly of the viral replication complex, lining the interior of the replication spherule, and re-direction of host resources for the synthesis of viral progeny (2–6). UVs use −1 ribosomal frameshifting (−1 PRF) to extend the ORF1 protein into ORF2, which generates the full-length RNA-dependent RNA polymerase (RdRp) (7). Downstream of the RdRp ORFs is an intergenic region of various lengths that can contain an XRN 5ʹ to 3ʹ exonuclease termination site (8), followed by two overlapping ORFs. ORF3 encodes an intrinsically disordered protein involved in long-distance movement (9), and protection from nonsense-mediated decay (10), and ORF4 encodes a structured cell-to-cell movement protein (MP) in the expansive class of MPs known as 30K MPs (named after the molecular mass of the first identified MP in tobacco mosaic virus [TMV]). 30K MPs are found in numerous families of DNA and RNA plant viruses and are characterized by a prominent β-barrel jelly roll domain composed of 8 anti-parallel β-strands. Since this jelly roll fold is also present in many capsid proteins, it has been proposed that 30K MPs arose in early plant viruses, following the duplication of a capsid protein ORF and neofunctionalization of one of the paralogs (11). UV ORFs 3/4 are translated from a single sgRNA, with the ORF4 start codon located 13 nt downstream of the ORF3 start codon. The helper virus provides capsid proteins for UV encapsidation and a suppressor of RNA silencing (9), both of which are necessary for robust UV infection and aphid-mediated transmission to additional plants (12).

The recent use of metagenomics to detect viruses in symptomatic and asymptomatic crops/native plants has led to the discovery of unusual viruses with UV-like RdRps known as umbra-like viruses (ULVs) (Fig. 1B and C). ULVs subdivide into two groups based on their RdRp amino acid sequences, with Group 2 ULVs further divided into three classes (Fig. 1C) (13). ULVs and UVs share a common genomic organization of ORFs 1 and 2, and ULV RdRp synthesis also requires −1 PRF (14). In contrast with UVs, ULVs do not encode overlapping ORFs 3/4, and, with one exception, ULVs do not contain an intergenic region after ORF2 (Fig. 1B and C). Rather, the third ORF, if present, overlaps out-of-frame with the 3ʹ terminal region of ORF2. For Group 2/Class 2 citrus yellow vein-associated virus CY2 (previously known as CYVaV), this third ORF (p22) was identified as encoding a capsid-like protein with a β-barrel jelly roll structure that supports, but is not required for, systemic movement (15). A fourth ORF found in monocot-infecting Group 2/Class 2 ULVs is nearly completely embedded within ORF3 and encodes intrinsically disordered proteins of different sizes (13).

Unexpectedly, several Group 2 ULVs, including CY2-related CY1, only contain ORFs 1 and 2, and yet are capable of vascular-restricted systemic infection without a helper virus (15, 16). Movement in the absence of encoded MPs by CY1, CY2, and probably all other Group 2/Class 2 ULVs was proposed to be mediated by phloem protein 2 (PP2) (15), a highly conserved family of RNA-binding proteins that also mediate movement of some viroids (17, 18). Other ULVs have zero, one, or two additional ORFs of unknown origins that encode proteins with unknown functions.

Although knowledge is accumulating for Group 2/Class 2 ULVs CY1 and CY2, no Group 1 ULV has been investigated to date. Group 1 ULVs are more closely related to UVs and have been identified in *Ageratum conyzoides* (goat weed) in China (19), *Thuja orientalis* (arborvitae) in Korea (20), *Paspalum distichum* in China (21), *Panicum virgatum L*. (switchgrass), and grapevine in Crimea (22). Of these Group 1 viruses, only ageratum ULV (AgULV), the most closely related to UVs, was identified in plants along with a presumptive helper virus from the enamovirus genus (19). Here, we report the first detailed examination of Group 1 ULVs, including a phylogenetic analysis of UV- and ULV-encoded proteins. We provide evidence that ULVs evolved from UVs following loss of ORF3, duplication of the 30K MP ORF 4, and neofunctionalization of one paralog into a jelly roll capsid-like protein and the second into a partial jelly roll protein of unknown function. Since neither paralog was required for systemic infection of a grapevine ULV (GULV4), it is likely that GULV4 uses a host MP for systemic infection, similar to CY1 and CY2. Our examination of known or predicted RNA structures important for replication and translation of UVs and ULVs revealed new conserved elements at the UV frameshifting site, and that Group 1 ULVs are more closely related to UVs than to Group 2 ULVs. Most Group 1 ULVs contain a previously described translation enhancer near their 3ʹ end known as an I-shaped structure (ISS), which is not found in UVs or Group 2 ULVs. Several of these ISS are predicted to adopt a novel branched configuration with separate hairpins containing the conserved eukaryotic initiation factor (eIF)4F-binding motif and long-distance interacting sequence. These findings lay the groundwork for further examination of these highly unusual viruses.

## RESULTS AND DISCUSSION

### Identification of missing 5ʹ and 3ʹ ends and annotation errors in UV and ULV reported sequences

**Fig 2:**
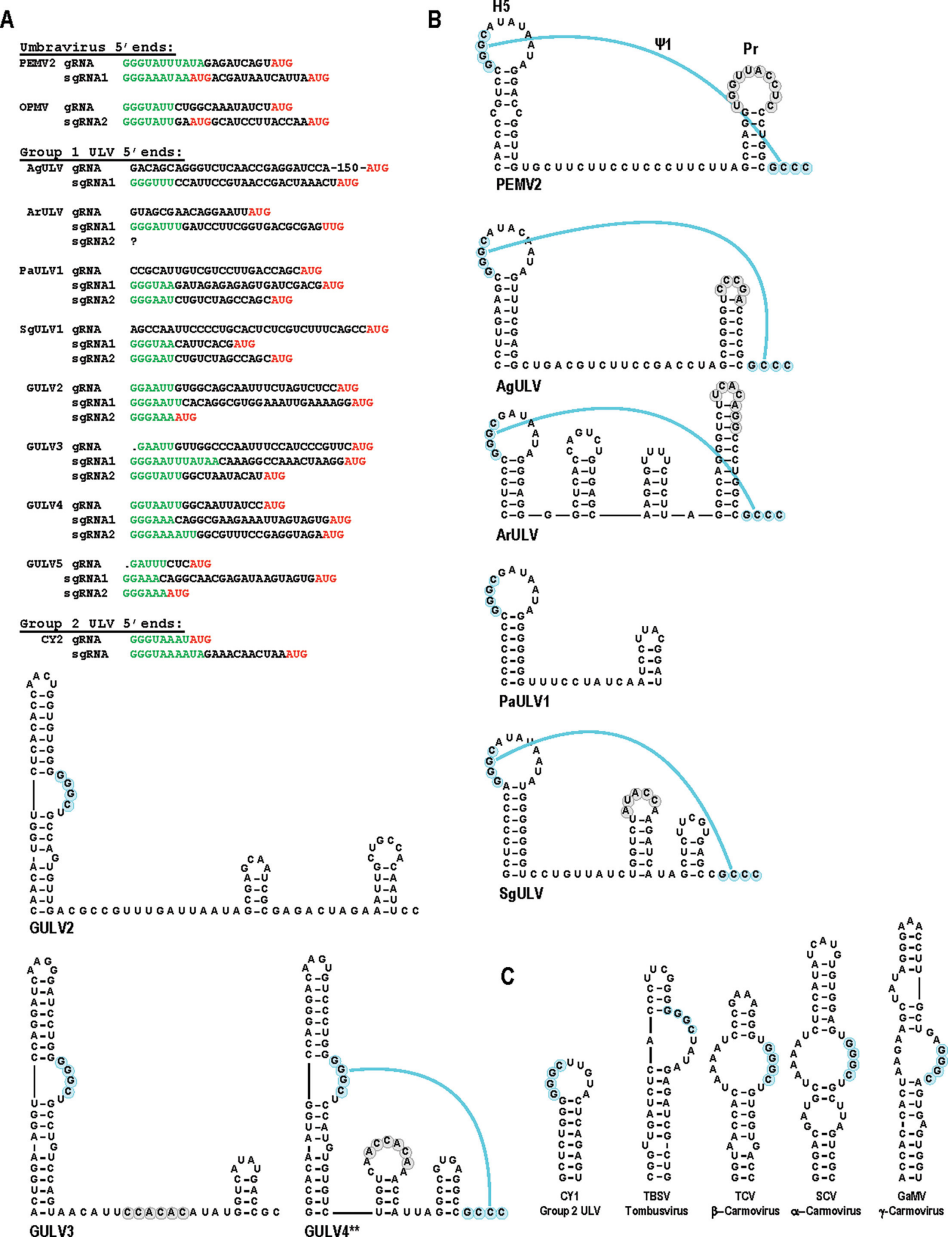
Sequences and structures at the 5ʹ and 3ʹ ends of UVs and ULVs. (**A**) Carmovirus consensus sequences (CCS; in green) are located at the 5ʹ ends of known UV and Group 2 ULV gRNAs and sgRNAs. Start codons are in red. Four Group 1 ULV gRNA sequences do not contain CCS at their 5ʹ ends and are likely truncated. SgRNA start sites can be predicted by the presence of a CCS just upstream of annotated start codons. ArULV p8 ORF does not contain an upstream CCS and may not be a genuine ORF. (**B**) Pseudoknot 1 (Ψ1; aqua) is located at the 3ʹ ends of UVs and carmoviruses. Group 1 ULVs missing this invariant structure are likely truncated. Residues shaded gray engage in the LDI with the FSE. The sequence shown for SgULV1 is followed by a duplication of residues in positions 3109–3170, which are omitted from the figure. **GULV4 denotes that the reported sequence contains only two 3ʹ terminal cytidylates, but sequences in infected plants contain three as shown here. (**C**) Examples of different H5 conformations. CY1, Group 2/Class 2 ULV CY1; TBSV, tomato bushy stunt virus (tombusvirus); TCV, turnip crinkle virus (β-carmovirus); SCV, saguaro cactus virus (α-carmovirus); and GaMV, galinsoga mosaic virus (γ-carmovirus).

Most UV and ULV sequences were selected for this study based on sequence length and the GenBank description of being full-length. To confirm whether sequences were indeed full-length, the 5ʹ ends of the genomic (g)RNAs were examined for the presence of a carmovirus consensus sequence (CCS) (23), comprised of G_2-3_A/U/C A/U_3-8_, which are known to be at the 5ʹ ends of gRNAs and subgenomic (sg)RNA of carmoviruses, UVs, and Group 2 ULVs (24–26) (Fig. 2A). Five “full-length” UV sequences are missing a 5ʹ CCS and thus are likely truncated (Table 1). Of the Group 1 ULVs, the four grapevine isolates (GULV2-5) contain canonical CCS at their 5ʹ ends (or lack a single G). The single available AgULV sequence is missing a 5ʹ CCS, and its ORF1 protein, which is annotated to be at least 50% shorter than ORF1 proteins of UVs and other ULVs, likely includes at least the 59 upstream amino acids. The single sequence available for arborvitae ULV (ArULV) is missing a 5ʹ CCS and its ORF1 is significantly longer (1.5-fold to 2.5-fold) than ORF1 for any other UV or ULV, suggesting possible issues with the reported sequence. Switchgrass ULV (SgULV1) is missing a 5ʹ CCS, and thus, designation of the ORF1 initiation codon is tentative. Guiyang Paspalum paspaloides tombus-like virus 1, which we will refer to here as PaULV1, is also missing a 5ʹ CCS but has an in-frame termination codon just upstream of the annotated ORF1 initiation codon. Therefore, the PaULV1 ORF1 annotation is likely correct.

**TABLE 1:** Presence of replication and translation elements in UVs and ULVs^*a*^^,^^*g*^

Name	Ψ1	TSS	3ʹCITE	LDI (5ʹ g or sgRNA-3ʹCITE)	CAS	CAS +KL	Annotation Issues
Umbravirus							
CMoV	Yes	Yes	UTE	GAUCA-UGAUC	Yes	No	FL^*b*^^,*e*^
PasUV	Yes	Yes	UTE	GAUCA-UGAUC	Yes	Yes	FL^*c*^^,*f*^
WCMoV	Yes	No	UTE	CUCUGG-CCAGAG	Yes	No	FL^*d*^
CMoMV	Yes	No	UTE-like	UAACCAG-CUGGUUA	Yes	No	FL^*b*^
CUV	Yes	No	UTE	UCACC-GGUGA	Yes	No	FL^*c*^^,*e*^
PicUV	?^*i*^	Yes	BTE-B	UGAC-GUCA	Yes	Yes	FL^*b*^^,*e*,*f*^
IxYMoV2	?	?	BTE-B	?	Yes	Yes	FL^*c*^^,*e*^
PMMoV	Yes	Yes	BTE-B	UGACU-AGUCA	Yes	Yes	FL^*b*^
WSVA	Yes	Yes	BTE-B	GACUA-UAGUC	Yes	Yes	FL^*d*^
PEMV2	Yes	Yes	KL-TSS+PTE	**UGGC**GA-UC**GCCA**	Yes	Yes	FL^*d*^
RCUV	Yes	Yes	New-unique	**UGGC**G-C**GCCA**	Yes	Yes	PS^*d*^
CJUV	Yes	Yes	UTE	**UGGC**A-U**GCCA**	Yes	Yes	PS
ETBTV	Yes	Yes	BTE-A	UCAACA-UGUUGA	Yes	Yes	FL^*d*^
GRV	Yes	Yes	BTE-A	GUUCA-UGAAC	Yes	Yes	FL^*d*^^,*f*^
PaeUV	Yes	Yes	BTE-A	**GCCA**AC-GU**UGGC**	Yes	Yes	FL^*b*^^,*f*^
OPMV	Yes	Yes	BTE-A	**UGGC**A-U**GCCA**	Yes	Yes	FL
TBTV	Yes	Yes	BTE-A	GGUUA-UAACC	Yes	Yes	FL
Group 1 ULVs							
AgULV	Yes	Yes	ISS	G**GCCA**A-U**UGGC**C	Yes	Yes	FL^*b*^
ArULV	Yes	No	New-unique	?	Yes	Yes	FL^*c*^
PaULV1	Yes	No	ISS	GG**GCCA**G-C**UGGC**CC	No	No	PS^*b*^^,*e*^
SgULV1	Yes	No	ISS	**GCCA**A-U**UGGC**	No	No	FL^*b*^^,*f*^
GULV2	?	No	ISS	**UGGC**AGC-GCU**GCCA**	No	No	FL^*e*^
GULV3	?	No	ISS	U**UGGC**UA-UA**GCCA**A	No	No	FL^*b*^^,*e*^
GULV4	Yes^*h*^	No	ISS	**UGGC**GA-UC**GCCA**	No	No	FL^*e*^
GULV5	?	No	ISS	U**UGGC**U-A**GCCA**A	No	No	PS^*b*^^,*e*^
Group 2 ULV							
CY1	Yes	No	ISS-like	?	No	No	FL

^*a*^ Virus ordering is according to Fig. 1D, and virus abbreviations and accession numbers are defined and shown in Table S1. Sequences in bold/underlined are a common UV and carmovirus long-distance RNA:RNA interaction sequence.

^*b*^ 5ʹ end likely truncated.

^*c*^ 5ʹ end has extra nucleotide(s).

^*d*^ ORF3 initiation codon is 5ʹ of sgRNA start.

^*e*^ 3ʹ UTR truncated.

^*f*^ 3ʹ end has extra nucleotide(s).

^*g*^ FL, virus entry described as full length; PS, virus entry described as partial sequence; LDI, long-distance interaction; TSS, T-shaped structure 3ʹCITE; CITE, cap-independent translation enhancer; CAS, CITE-associated structure; KL, kissing loop; RSE, recoding stimulatory element.

^*h*^ Missing 3ʹ cytidylate is present in GULV4 in infected plants.

^*i*^ ? denotes that presence of element cannot be determined.

The four 3ʹ terminal residues of UVs and related viruses in the *Tombusviridae* participate in a distinctive pseudoknotted structure termed ψ1 (27, 28). These 3ʹ residues, usually GCCC-OH, are located just downstream of the 3ʹ terminal hairpin (named “Pr”). The ψ1 partner residues (5ʹ-GGGC) are located in a nearby upstream hairpin (termed “H5”) (13, 29) (Fig. 2B). All UV and Group 2 ULV hairpin H5s are simple hairpins with the GGGC located on the 5ʹ side of the conserved apical loop. This H5 structure is unique among the *Tombusviridae*, which otherwise contain ψ1 residues at the 3ʹ side of a symmetrical or asymmetrical internal loop (Fig. 2C). Residues in the region encompassing H5 and Pr also participate in a critical long-distance RNA:RNA interaction (LDI) with sequences in the upstream frameshift stimulatory element (FSE), which is located just downstream of the ORF1 stop codon (7, 14, 30). Therefore, a complete ULV 3ʹ sequence should have a terminal GCCC-OH motif, a corresponding H5 hairpin, an intervening Pr hairpin, and ψ1.

Group 1 AgULV and ArULV sequences terminate with a UV-type ψ1 and H5, and both have the FSE LDI sequence in the apical loop of Pr, typical of UVs (Fig. 2B). The SgULV1 sequence also has canonical H5, Pr, and ψ1, with the LDI sequence in the apical loop of the penultimate hairpin, similar to Group 2 ULVs. This suggests that these sequence entries have complete 3ʹ ends. In contrast, the PaULV1 sequence is missing the 3ʹ terminal residues needed for ψ1 and is likely truncated. Although the GULV isolates contain an H5 and ψ1, the H5 is in an unusual conformation with the ψ1 sequence in an asymmetric internal loop (similar to that of tombusviruses) but without the opposing adenylate residue in the internal loop (Fig. 2B and C). In addition, GULV5 contains a 3ʹ end truncation just past H5, whereas GULV2-4 are missing one or two cytidylates at their 3ʹ ends that would be necessary to form a canonical ψ1. As described below, GULV4 gRNA in systemically infected *Nicotiana benthamiana* terminated with three cytidylates; thus, Group 1 GULVs likely also contain ψ1.

UV ORFs 3/4 are translated from one sgRNA with a CCS at the 5ʹ end (8, 24). However, many UV sequences have annotated ORF3 initiation codons upstream of the CCS (Table 1). Since CCS are at the 5ʹ ends of sgRNAs, it is likely that these ORF3 annotations have inaccurate N-terminal extensions added to the encoded proteins. Group 1 ULVs (except for putative ArULV p8 ORF4) have canonical CCS just upstream of all annotated ORF3s and ORF4s (Fig. 2A), suggesting that these ORFs encode proteins expressed from individual sgRNAs. In contrast, some Group 2/Class 3 ULV ORFs 3/4 are annotated as separate ORFs (31) but may instead be joined through ribosomal readthrough of the ORF3 termination codon (13). This proposed revised genomic organization was based on the lack of a CCS upstream of ORF4, in-frame positioning of the two ORFs with no intervening in-frame stop codons, and the presence of structures just past the ORF3 termination codon that strongly resemble those at readthrough recoding sites in carmoviruses. Since ArULV ORF4 is not preceded by a CSS, its presence as a bona fide ORF is in question.

### Phylogenetic relationships among UV and ULV non-replicase ORFs

As described above, UVs and ULVs differ significantly in the locations of their non-replicase ORFs (Fig. 1A through C). UV ORFs 3/4 are embedded and downstream of an intergenic region, whereas ULVs have zero, one, or two additional ORFs, with ORF3 overlapping the 3ʹ end of ORF2, with the exception of AgULV. Of the ULVs with four ORFs, ORF3 and ORF4 either overlap nearly completely (monocot-infecting Group 2/Class 2 ULVs), overlap partially (Group 1 non-grapevine ULVs), or do not overlap (GULV2-5) (Fig. 1B and C).

**Fig 3:**
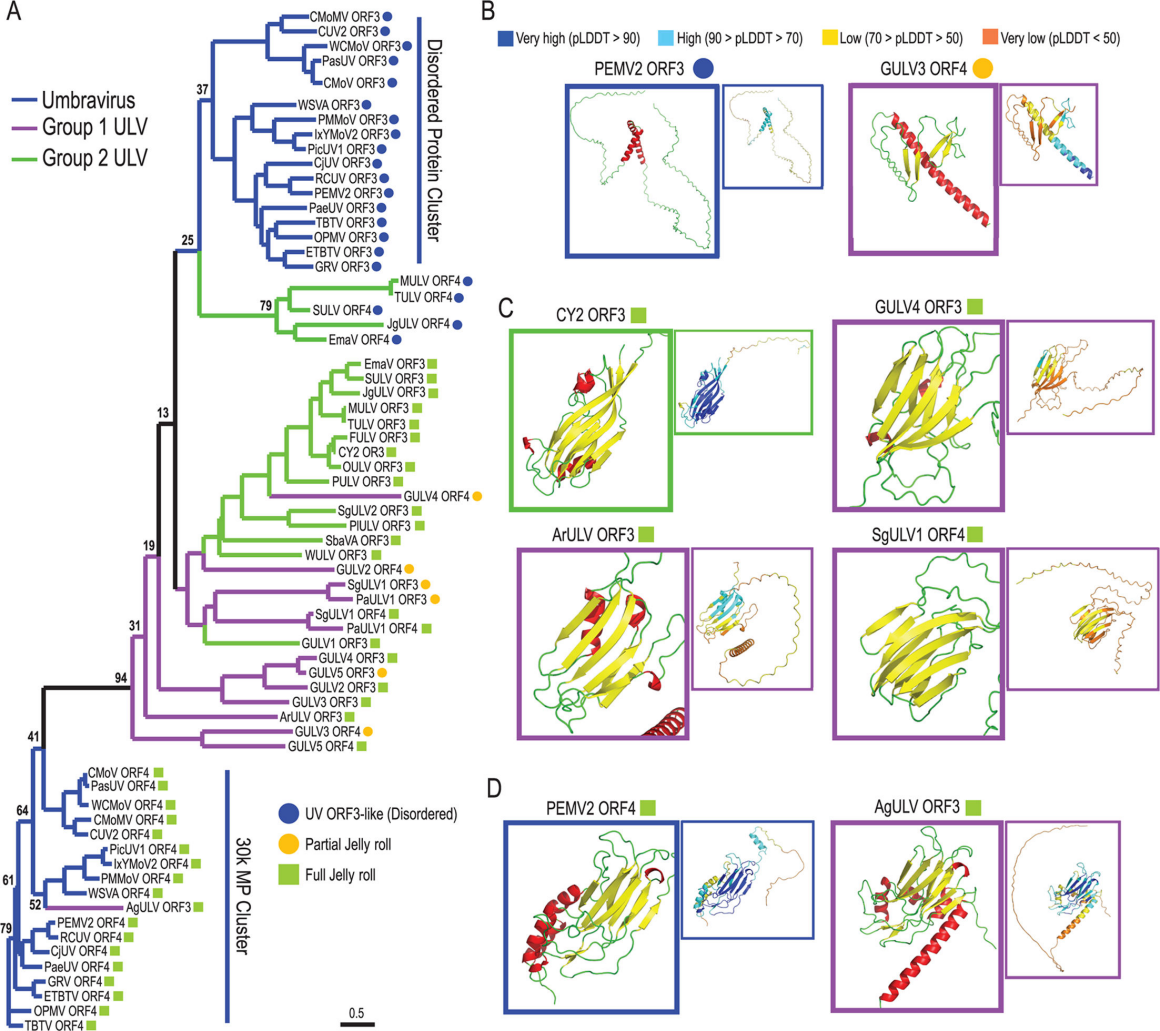
Phylogenetic and structural analyses of ULV ORF3 and ORF4 proteins. (**A**) Maximum-likelihood phylogenetic tree based on ORF3 and ORF4 amino acid sequences out of 1,000 replicates. Bootstrap support values for the major clade nodes are indicated. All ORFs were first checked for an upstream CCS prior to running the alignment. ULV sequences are marked with a green square if the AlphaFold3-predicted structure contains at least seven β-strands in a jelly roll fold. ULV ORF-encoded proteins with less than seven β-strands were labeled as partial jelly roll proteins and are denoted with an orange circle. The scale bar represents the number of amino acid substitutions per site. (**B**) AlphaFold3 predictions of disordered PEMV2 ORF3 and Group 1 GULV3 partial jelly roll protein (ORF4). (**C**) Structure predictions of full jelly roll proteins from Group 2/Class 2 CY2 ORF3, and Group 1 ULV GULV4 ORF4, ArULV ORF3, and SgULV1 ORF4. (**D**) Structure predictions for PEMV ORF4 30K MP and AgULV ORF3 full jelly roll protein. Small boxes show confidence levels for the predicted structures; structures in large boxes are colored with α-helices in red, β-strands in yellow, and the remainder in green. The 3D structure prediction files in PDB format for all structures can be found in Data set S2.

UV overlapping ORFs 3/4 encode MPs with very different structures. UV ORF3 proteins are predicted by the state-of-the-art, high-performance structural modeling program Alphafold3 (32) to be highly variable with large stretches of intrinsically disordered regions and no common motifs other than a positively charged N-terminus (Fig. 3B). The ORF4-encoded protein is identifiable as a 30K MP by its distinctive full jelly roll fold (7–8 antiparallel β-strands) containing a D-motif between the E and F β-strands (Fig. 3D) (11). We recently reported that ORF3 of Group 2/Class 2 CY2 (previously named ORF5) was predicted by Alphafold3 to encode a protein with a full jelly roll (Fig. 3C) (15). However, the CY2 ORF3 protein differed from the UV 30K MP in distinctive ways. First, the N-terminal domain of the CY2 ORF3 protein contained a long, intrinsically disordered region with a nucleolar localization signal. Second, alignment with 30K MP amino acid sequences suggested that the CY2 ORF3 protein lacked the D-motif that is ubiquitous in 30K MPs. Third, CY2 ORF3 supported but was not required for systemic movement, unlike 30K MPs. Since the presence of the CY2 ORF3 protein was also associated with virion-like structures in infected plants, this protein was labeled as a jelly roll-containing capsid-like protein (15).

Given the proposed origin of 30K MPs from capsid proteins (and not vice-versa) (11), it was suggested that the CY2 capsid-like protein did not evolve from UV 30K MPs but rather was acquired through horizontal acquisition from another virus via recombination events thought to occur frequently during plant virus evolution (11, 15). To determine whether a recombination event had occurred with a virus outside of the UV/ULV lineage, we compared the predicted structures and sequences of UV and ULV ORFs 3/4. Due to frequent mis-annotations of UV and ULV ORF 3/4 start positions in reported sequences as described above, ULV ORFs 3/4 were reannotated to consider the presence of an upstream CCS and named based on their order in the genome, with one exception. ORFs predicted to encode a jelly roll protein similar to CY2’s capsid-like protein in dicot-infecting and monocot-infecting Group 2/Class 2 ULVs were both labeled as ORF3. Correspondingly, the embedded and variable-sized ORFs in monocot-infecting Group 2/Class 2 were labeled as “ORF4” despite the embedded proteinʹs initiation codon being two codons upstream of the ORF3 initiation codon.

All ULV ORF 3/4 proteins were subjected to folding by Alphafold3. Interestingly, either ORF3 or ORF4 in all ULVs encoded a protein that was predicted to have a full jelly roll fold (defined here as having at least seven anti-parallel β-strands). For Group 1 ULVs, the remaining ORFs (if any) encoded a protein predicted to have a subset of β-strands along with more disordered regions and thus were termed a “partial jelly roll protein” (Fig. 3B through D). This contrasts with the embedded ORF4 in Group 2/Class 2 ULVs, which were not predicted to contain any consistent motifs. While comparing the predicted structures of the full jelly roll proteins, we observed that the β-strand orientation differed from that of the UV ORF4 30K MP. Specifically, the E and F strands of the ULVs were further downstream in the C-terminus, whereas the 30K MP E and F strands were closer to the N-terminus (Fig. S1A). Given this difference, a simple sequence alignment would miss the presence of a D-motif. Indeed, we observed that the full jelly roll proteins, including the re-analyzed CY2 ORF3 protein, contain aspartic acid residues (the D-motif) between the E and F strands typical of a D-motif (Fig. S1B). Therefore, it is important to consider not only simple sequence alignment but also protein structure for determining the presence of a D-motif.

If ULV ORFs 3/4 arose from a recombination event with one or more unrelated viruses, they should form out-groups from UV ORFs 3/4 in a phylogenetic analysis. To test this, we aligned the amino acid sequences of UV and ULV ORF 3/4 proteins and generated a phylogenetic tree (Fig. 3A). As expected, UV ORF3 and ORF4 proteins clustered into separate clades corresponding to either an embedded, disordered cluster (UV ORF3s) or the 30K MP cluster (UV ORF4s). The full jelly roll ORF3 protein of AgULV clustered among the UV 30K MPs and was predicted to contain a similar conformation of the β-strands and a 30K MP D-motif (Fig. 3D; Fig. S1C). The GULV5 ORF4 full jelly roll protein and GULV3 ORF3 partial jelly roll protein were the next most closely related to UV ORF4 30K MPs, with the GULV5 full jelly roll protein also having a UV D-motif (Fig. S1C). These close relationships suggest that both AgULV ORF3 and GULV5 ORF4 proteins are descended from the UV ORF4 30K MP rather than being introduced by a recombination event with an unrelated virus. Indeed, nucleotide alignment of UV and ULV ORFs 3/4 shows that GULV5 ORF4 and AgULV ORF3 nucleotide sequences cluster together alongside carrot-infecting UV ORF4 sequences (Fig. S2), supporting the proposal that these full and partial jelly roll proteins evolved from the UV 30K MP. In addition, the close relationship between some ULV ORF3 and ORF4 nucleotide sequences (e.g., PaULV1 and SgULV1) strongly suggests that these ORFs were generated by a duplication event (Fig. S2).

The remaining ULV ORFs varied in their relationships with the UV ORFs, rather than forming a distinct out-group. Disordered, embedded Group 2/Class 2 ORF4 proteins clustered with the disordered embedded UV ORF3 clade (Fig. 3A). Most Group 1 ULV partial jelly roll proteins clustered with Group 1 ULV full jelly roll proteins, close to the UV 30K MP clade. The exception, GULV4 ORF4 partial jelly roll protein, clustered with Group 2 ULV full jelly roll proteins (i.e., CY2 ORF3). Altogether, these relationships do not support a model where ULV ORFs arose from a recombination event with an unrelated virus. Rather, the relationships suggest a common origin for Group 1 ULV ORFs 3/4 and Group 2 ORF3 from the 30K MP-encoding ORF4 of UVs.

### Predicted higher-order conformations of known 30K MPs differ from ULV full jelly roll proteins and the full jelly roll capsid protein of barley yellow dwarf virus

**Fig 4:**
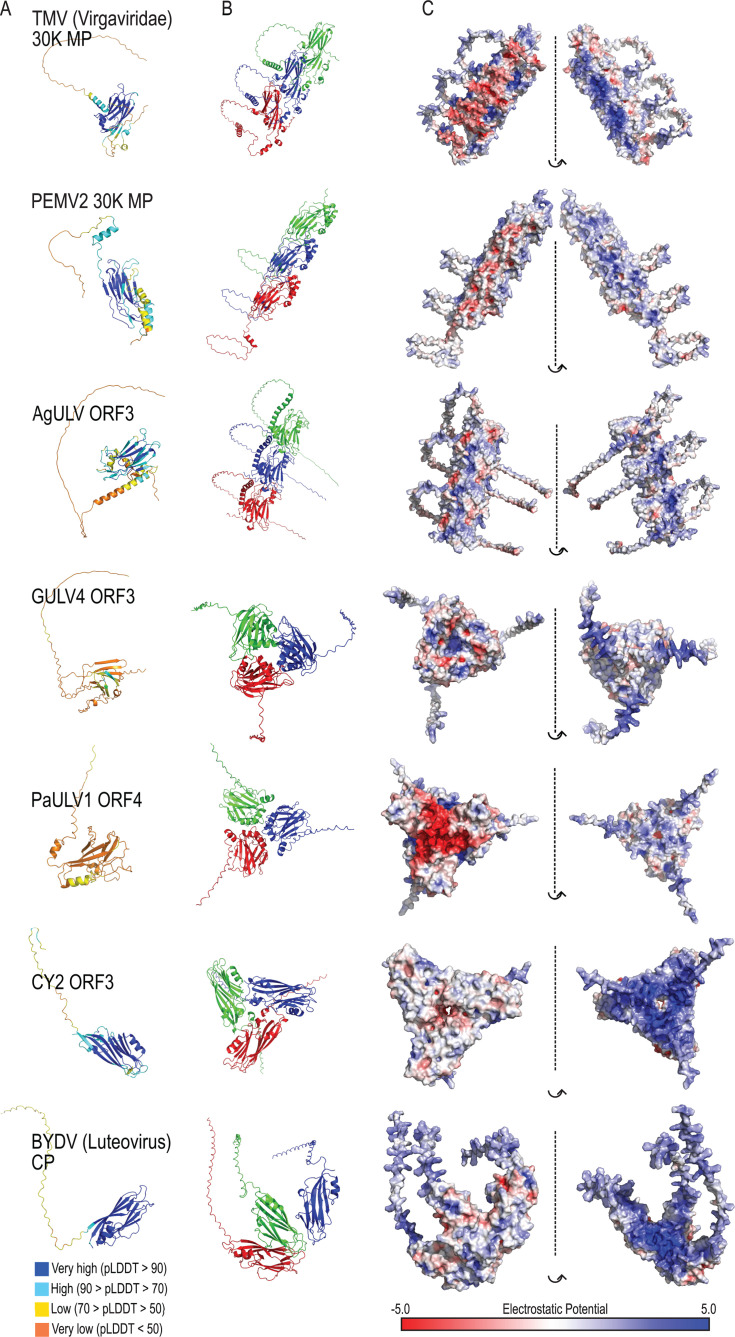
Predicted higher-order structures of 30K MP and ULV full jelly roll protein multimers. (**A**) Alphafold3-predicted structure of the indicated protein monomers. Residues are colored based on the predicted confidence. (**B**) The predicted higher ordered structures of homotrimers of the proteins from (**A**). Individual monomers are colored red, blue, or green. (**C**) Surface models of the higher ordered structures predicted in (**B**) are colored with the estimated electrostatic potential. Blues, positively charged; reds, negatively charged. These predicted structures, as well as the structural predictions of higher-order complexes, have been included at Figshare DOI: https://doi.org/10.6084/m9.figshare.31281964.

While we have shown that CY2 ORF3 encodes a capsid-like protein and not a traditional 30K MP (15), it is unknown whether the full jelly roll proteins of Group 1 ULVs are more similar to capsid-like proteins or 30K MPs. Some 30K MPs, including the UV 30K MPs, are known to organize into tubule-like structures *in vivo* (33–35), whereas capsid proteins use an ordered assembly process to form discrete transient intermediates (36). This suggested to us that the function of jelly roll proteins may be revealed by examining predicted higher-order structures. To determine if higher-order assembly of 30K MP monomers differs from those of ULV full jelly roll proteins, trimers of selected proteins were modeled using Alphafold3 (Fig. 4). The 30K MPs from TMV (family *Virgaviridae*), UV PEMV2, and ULV AgULV all assume an extended linear “building block” conformation consistent with extended tubular formation. In contrast, barley yellow dwarf virus (BYDV; family *Tombusviridae*) capsid protein, which structurally resembles the CY2 ORF3 capsid-like protein (15), did not form tubular-type structures. Rather, BYDV CP trimers appeared to form half of a hexon, typical of an icosahedral virion, with a continuous positive charge across one plane of the structure (Fig. 4C). Trimers of ULV full jelly roll proteins from GULV4 (ORF3), PaULV1 (ORF4), and CY2 (ORF3) formed trimeric structures that were also significantly different from the 30K MP tubule structures. Since the higher-order structures of these Group 1 full jelly roll proteins were similar to the higher-order structure formed by the CY2 capsid-like protein, these results support our model that, with the exclusion of AgULV, ULV full jelly roll proteins have different properties from 30K MPs and are likely capsid-like proteins.

### Model for the evolution of ULVs

**Fig 5:**
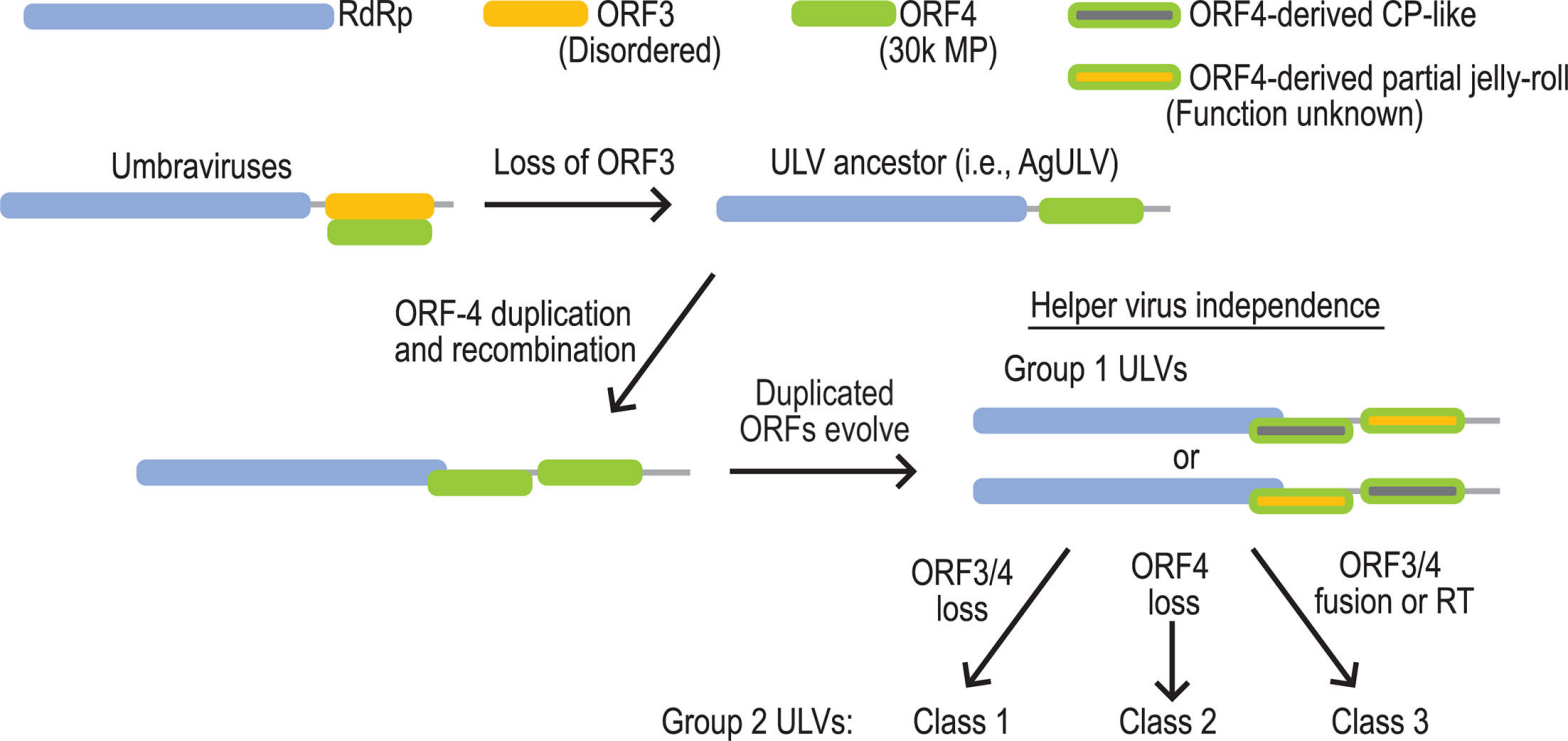
Model of ULV evolution from UVs. UVs encode both a 30K MP (ORF4) and embedded, disordered protein (ORF3). We propose that an AgULV-like ULV arose following the loss of ORF3 coding capacity. This new virus would have maintained the need for a helper virus for encapsidation, suppression of RNA silencing, and possibly long-distance movement. We propose that duplication of ORF4 in the AgULV-like ancestor included recombination into the RdRp coding region at amino acid residues whose codons comprise a CCS. Free of the genetic constraint of an embedded protein, one of the ORF4 paralogs diverged to encode a capsid-like protein, with the other paralog evolving to encode a partial jelly roll protein of unknown function. This would have removed the requirement for a helper virus for plant-to-plant transmission and required the use of an endogenous RNA transport protein like PP2 for systemic movement. Further evolution and/or loss of the neofunctionalized ORFs would then give rise to the various Group 2 ULV classes.

We propose a model whereby an ancestor ULV (similar to AgULV) evolved from UVs following the loss of embedded ORF3 (Fig. 5). This ancestral virus would still encode the 30K MP and, similar to AgULV, would likely retain the requirement for a helper virus. The loss of ORF3 would have required the evolution of a new type of long-distance movement support. Since ORF3 and ORF4 of Group 1 ULVs cluster together in both the amino acid and nucleotide phylogenetic trees, contain full or partial jelly roll structures, and the full jelly roll protein is encoded by either ORF3 or ORF4, we propose that the 30K MP ORF was duplicated in an event that also removed the intergenic region. Now free of the genetic constraint of an embedded protein, one of the 30K MP paralogs acquired a different function as a capsid-like protein while retaining the full jelly roll structure. Since ULVs (with the exception of AgULV) are reported to be in plants without a helper virus, evolution of a capsid-like protein could have allowed for independent encapsidation and vector-mediated transmission to new plants. If the capsid-like protein was able to support vascular movement mediated by a host-encoded MP, the other paralog was free to evolve into a partial jelly roll protein of unknown function. Loss of the downstream paralog ORF is proposed to have generated Group 2/Class 2 dicot-infecting ULVs, whereas the reappearance of an embedded, disordered ORF4 within ORF3 would have generated Group 2/Class 2 monocot-infecting ULVs. Group 2/Class 1 ULVs, which only contain ORFs 1/2 and are restricted to plants that propagate vegetatively (16), could have resulted from the loss of both paralog ORFs.

### Group 1 GULV4 ORFs 3/4 are not required for systemic infection of *N. benthamiana*

**Fig 6:**
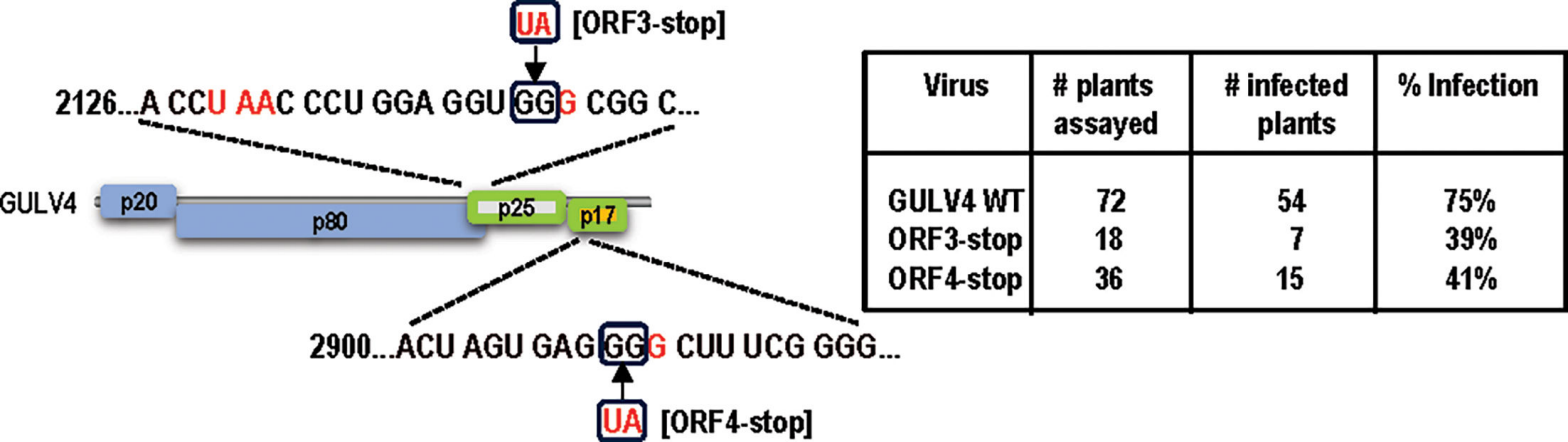
GULV4 is capable of systemic infection in the absence of ORF3 and ORF4. Left, diagram showing the location of premature stop codons engineered in ORF3 (ORF3-stop) or ORF4 (ORF4-stop). Since ORF3 overlaps with ORF2, G2143U and G2144A mutations were generated downstream of the ORF2 stop codon (red). Right, the results of infection of *N. benthamiana* with wild-type GULV4 (WT), ORF3-stop, or ORF4-stop. Plants were agro-infiltrated using vacuum infiltration along with silencing suppressor p19 from tomato bushy stunt virus (TBSV). Positive systemic infections were determined by RT-PCR of RNA extracted from systemic leaves at 2 weeks post-infiltration. The percentage of infected plants and the total number of plants tested are shown.

The full jelly roll capsid-like protein encoded by CY2 ORF3 is not required for systemic infection of *N. benthamiana* but shortens the timing to first symptoms (15). If our model is correct, and ULVs other than AgULV no longer encode an MP, then the full jelly roll capsid-like proteins of Group 1 ULVs should also be dispensable for systemic infection. To investigate this possibility, a full-length clone of Group 1 GULV4 was generated from the reported sequence, which terminated in two cytidylates rather than the canonical three cytidylates of UVs, Group 2 ULVs, and other Group 1 ULVs. Two weeks following *Agrobacterium tumefaciens-*mediated infiltration of wild-type (WT) GULV4 into *N. benthamiana*, systemic leaves were assayed for the presence of GULV4 gRNA using RT-PCR. Seventy-five percent of the infiltrated plants (54 of 72) were positive for GULV4 (Fig. 6B; Fig. S3B). Infiltrated leaves of GULV4-positive plants contained a distinctive necrosis (Fig. S3A). However, unlike the severe stunting and leaf-curling symptoms on systemic leaves of CY1- and CY2-infected *N. benthamiana*, GULV4 infection did not elicit systemic symptoms. Despite initiating infection with only two 3ʹ terminal cytidylates, direct RNA nanopore sequencing of total RNA from one infected plant revealed that all GULV4 RNA reads contained the canonical three cytidylates at their 3ʹ ends. This strongly suggests that the reported Group 1 GULV sequences may be missing their 3ʹ terminal residues.

To determine if ORF3 and/or ORF4 are necessary for infection of GULV4, termination codons were individually engineered downstream of ORF3 and ORF4 initiation codons, generating ORF3-stop and ORF4-stop, respectively (Fig. 6, left). At 2 weeks following agro-infiltration, GULV4 was detected in 39% of ORF3-stop plants and 41% of ORF4-stop plants (Fig. 6, right; Fig. S3B). Similar to infection with WT GULV4, only the infiltrated leaves displayed necrotic symptoms. PCR bands amplified from ORF3-stop- and ORF4-stop-infected plants were subjected to batch sequencing, which confirmed retention of the mutations (Fig. S3C). These results indicate that ORF3 and ORF4 are not required for systemic movement of GULV4 in *N. benthamiana*, supporting our model that GULV4 is using a host MP, similar to Group 2 ULVs CY1 and CY2 (15).

### UVs and Group 1 ULVs share similar structures and sequences near the frameshift recoding site that differ significantly from those of Group 2 ULVs

To further define the relationship among UVs, Group 1 ULVs, and Group 2 ULVs, RNA elements involved in replication and translation previously identified for UVs PEMV2 and OPMV and Group 2/Class 2 ULV CY1 were examined for all 17 UV and 8 Group 1 ULVs.

**Fig 7:**
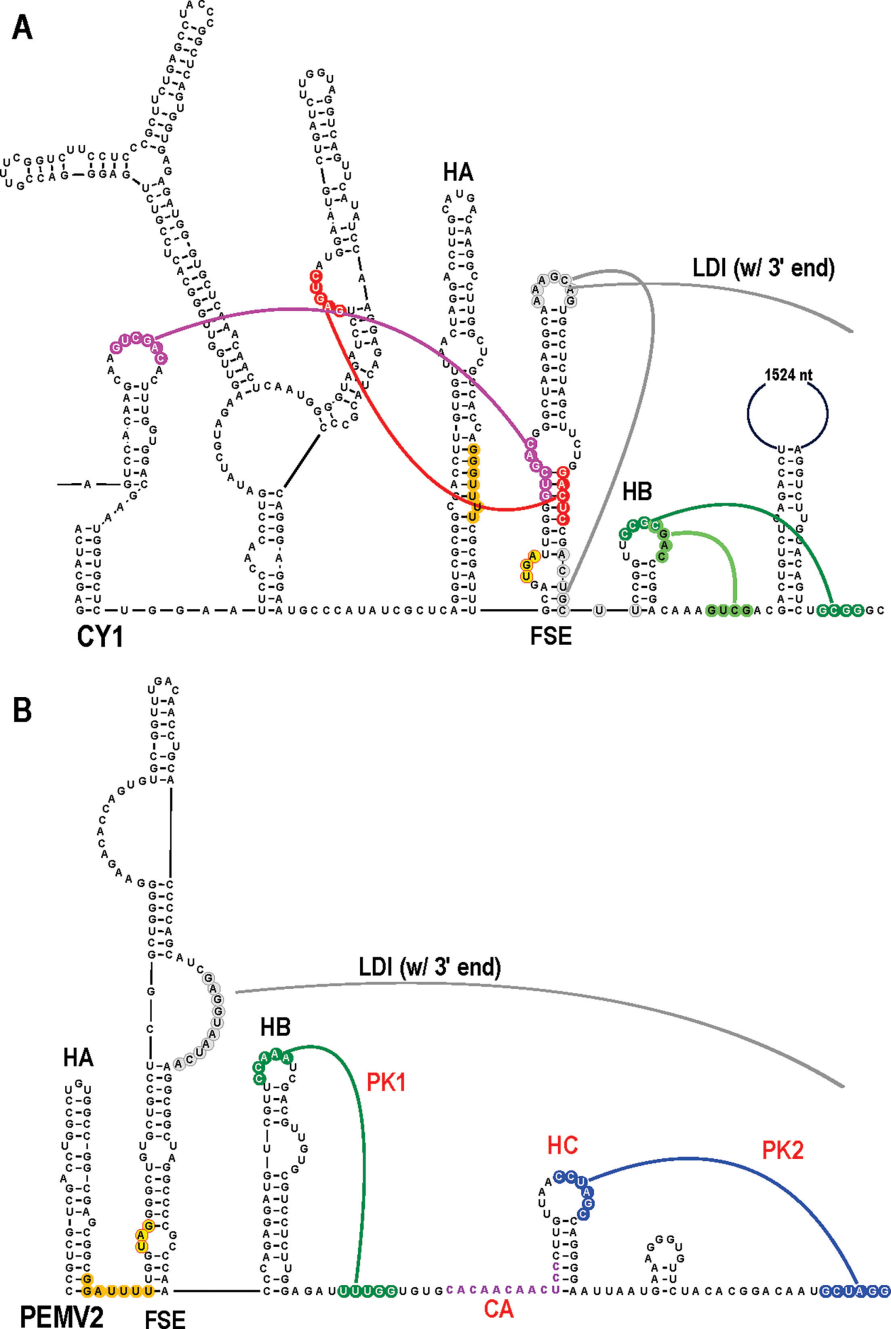
Comparison of ribosome frameshifting sites for Group 2/Class 2 CY1 and UV PEMV2. (**A**) Active structure of the CY1 frameshifting site. Known tertiary interactions are color-coded. Slippery sequence is in orange. ORF1 stop codon is in yellow. (**B**) Recoding site of UV PEMV2. Structures and interactions labeled in red were not previously known but are conserved in the majority of UVs (Table 2; Fig. S4).

The ribosome frameshifting sites for CY1 and PEMV2 consist of the following three hairpins (7, 14): (i) the frameshift stimulatory element (FSE); (ii) a hairpin upstream of the FSE (HA), which is analogous to the coronavirus attenuator sequence and is ubiquitous at ribosome recoding sites in the *Tombusviridae* (7, 14, 37); and (iii) a hairpin (HB) downstream of the FSE (Fig. 7). In CY1, FSE and HB connect to local and distal sequences by 6 RNA:RNA interactions, several of which are incompatible with a single conformation and only one of which, the LDI with the 3ʹ end, is found in PEMV2. In UVs, this latter LDI forms from sequences in an asymmetric internal loop of the FSE (Fig. 7B) (7). In Group 2 ULVs, the LDI sequences are in the FSE apical loop (Fig. 7A) (14). The location of the ribosome slippery sequence required for ORF2 also differs in UVs and Group 2 ULVs. In UVs, the sequence (mainly GGAUUUU) is located 0 or 3 nt upstream of the ORF1 termination codon, whereas in Group 2 ULVs, the sequence (mainly GGGUUUU) is located 6 or 12 nt upstream (Table 2; Fig. 6, orange).

**TABLE 2:** Elements associated with frameshifting sites in UVs and ULVs^*a*^

Name	Slippery site	PK1 sequence	PK2 sequence	CA at base of HC or in HC
Umbraviruses				
CMoV	*GGAUUUU*UGCUAG	ACGC-GCGU	None	In HC
PasUV	*GGAUUUU*UGCUAG	AUACCA-UGGUAU	None	In HC
WCMoV	*GGAUUUU*UGCUAG	None	CUCGU-ACGAG	HC base
CMoMV	*GGAUUUU*UGCUAG	AGAUU-AAUCU	None	In HC
CUV	*GGAUUUU*UGCUAG	None	CUCG-CGAG	HC base
PicUV	*GGGUUUU*CUGCUAG	CCCAA-UUGGG	CUCGU-ACGAG	HC base
IxYMoV2	*GGGUUUU*UGCUAG	CCCCA-UGGGG	None	HC base
PMMoV	*GGGUUUU*UGCUAG	CGAUG-CAUCG	AACA-UUGU	HC base
WSVA	*GGAUUUU*UGCUAG	GACGC-GCGUC	UCGUU-AACGA	HC base
PEMV2	*GGAUUUU*UGGUAG	CCAAA-UUUGG	CCUAGC-GCUAGG	HC base
RCUV	*GGAUUUU*UGGUAG	CCAAA-UUUGG	CCUGGC-GCCAGG	HC base
CJUV	*GGGUUUU*CACUAG	ACCA-UGGU	None	HC base
ETBTV	*AAAUUUU* UAG	UGUGGAC-GUCCACA	CUCAUU-AAUGAG	HC base
GRV	*AAAUUUU* UAG	UGUGGAC-GUCCACA	CUCAUU-AAUGAG	HC base
PaeUV	*GGAUUUU* UGA	AUUGAUGC-GCAUCAAU	None	HC base
OPMV	*GGAUUUU* UAA	AUCG-GCAU	UCAUUA-UAAUGA	HC base
TBTV	*GGAUUUU*UGCUAG	CCAAA-UUUGG	None	HC base
Group 1 ULVs				
AgULV	*GGGUUUU* UGA	None	None	HC base
ArULV	*GGGUUUU*CGGUAA	AUGCAC-GUGCAU	UUGC-GCAA	None
PaULV1	*GGAUUUU* UAA	CCAUAG-CUAUGG	UUGCG-CGCAA	HC base
SgULV1	*GGGUUUU*CGGUAG	GCCAUA-UAUGGC	None	HC base
GULV2	*GGGAAAC* UAA	UGUGG-CCACA	None	None
GULV3	*GGGUUUU* UAG	GUUGUG-CACAAC	None	HC base
GULV4	*GGGUUUC* UAG	UGUGG-CCACA	None	HC base?
GULV5	*GGGUUUC* UAG	UGUGG-CCACA	None	HC base?
Group 2 ULV				
CY1	*GGGUUUU*CGCGAUUUGCAGUGA	None	None	None

^*a*^ Virus ordering is according to Fig. 1D. Accession numbers are in Table S1. Slippery site is in italics; ORF1 stop codon is underlined. CA, consensus sequence CACAACAACUCC; HC, hairpin C; PK, pseudoknot.

The complex nature of Group 2 ULV frameshifting sites prompted a re-examination of the 17 UV recoding sites to determine if additional conserved features may have been missed. We found that 15 UVs, including PEMV2 and OPMV, contain 4–8 nt in their HB apical loops capable of forming Watson-Crick base pairs with nearby downstream sequences (labeled as “PK1” in Fig. 7B; see Table 2 for all PK1 sequences and Fig. S4 for all UV frameshifting site structures). This finding was surprising, as the HB apical loop was dispensable for frameshifting of full-length PEMV2 in *in vitro* assays using wheat germ extracts (7). However, deletion of the highly conserved HA hairpin also had no effect on frameshifting *in vitro*, suggesting that *in vitro* assays do not reflect *in vivo* requirements for PEMV2 frameshifting, as was found for other viral frameshifting sites (38). Since transitions between active and inactive RNA conformations at frameshifting sites are a hallmark of ribosome recoding (14, 39, 40), it is possible that PK1 is not required for the active frameshifting conformation. In addition to PK1, all 17 UVs have the consensus sequence CACAACAACUCC (termed “CA”) just downstream of PK1 (Fig. 7B, in purple), corresponding to a highly conserved “HNNS” amino acid motif. In 14 UVs, including PEMV2, the final 3–6 nt of the CA sequence form the lower stem of a hairpin, labeled HC in Fig. 7B. Additional sequences in the HC hairpin are not conserved, suggesting that the CA sequence upstream of a hairpin may constitute a frameshift site element rather than simply as codons for a conserved amino acid motif. For the remaining 3 UVs, the CA sequence is in the apical loop of a similarly located hairpin (Fig. S4A, B, and D). In addition to PK1, HC, and CA, 10 of 14 UVs with CA at the base of HC have a second possible Watson-Crick pseudoknot (labeled as “PK2” in Fig. 7B) between the HC apical loop and nearby downstream sequences (Table 2; Fig. S4).

Recoding region structures were predicted for all Group 1 ULVs to determine whether they conform more closely to those of UVs or Group 2 ULVs. All Group 1 ULV recoding sites contain HA and HB, and their FSEs conformationally resemble those of UVs, including a lower asymmetric loop that contains the 3ʹ end LDI sequence (Fig. S5). In addition, the location of the slippery site relative to the ORF1 termination codon was identical to UVs (0 or 3 nt upstream of the ORF1 stop codon, Table 2). Seven of the 8 Group 1 recoding sites contained PK1, and all contained hairpin HC. Two of the ULV sequences could form PK2, and at least four had a CA sequence upstream and forming the base stem of HC, as with most UVs (Table 2; Fig. S5). These observations suggest that Group 1 ULVs use a frameshifting mechanism more closely related to UVs than to Group 2 ULVs, supporting the closer evolutionary relationship between UVs and Group 1 ULVs.

### AgULV contains the structures necessary to form a UV-like T-shaped structure 3ʹCITE

**Fig 8:**
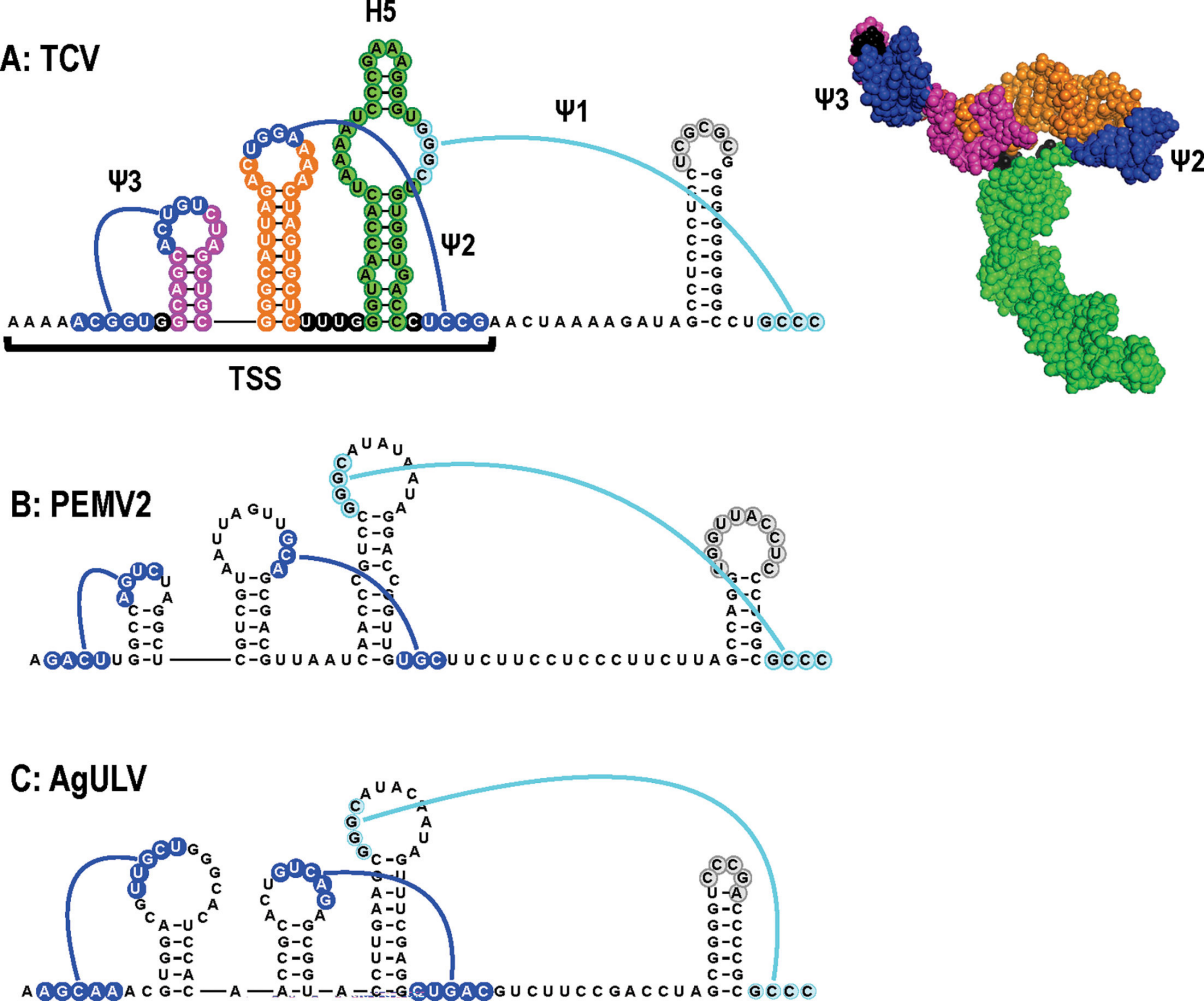
AgULV contains a TSS 3ʹCITE at its 3ʹ end. (**A**) 3ʹ terminal region of carmovirus TCV containing the first identified TSS. The TSS is composed of three hairpins and two pseudoknots and is color coded to match the structure at right, which was determined using NMR and SAXS (45). Additional coloring is according to Fig. 2B. Note that after the structure was solved, it was determined that the 5ʹ adjacent adenylates played an important role in strengthening the structure of Ψ3 (48). (**B**) TSS of PEMV2. (**C**) TSS predicted for AgULV.

A hallmark of members of the *Tombusviridae* is structures in their 3ʹ UTRs that support cap-independent translation from the 5ʹ ends of their coding RNAs. 3ʹ cap-independent translation enhancers (3ʹCITEs) either nucleate the 43S translation initiation complex by interacting with specific translation initiation factors or by directly binding to ribosomes (41). Most 3ʹCITEs connect with sequences at the 5ʹ end of the gRNA/sgRNA through LDIs that enhance translation without increasing the rate of initiation (42). 3ʹCITEs are generally divided into 7 or 8 classes based on their structures and conserved sequences, and different 3ʹCITEs can occupy identical locations within single genera, suggesting modular acquisition during evolution (41, 43, 44). Although plant viruses generally have only a single 3ʹ CITE, most UVs have two: one near the 3ʹ end and a second in the mid-section of their long (~700 nt) 3ʹ UTRs. The CITE near the 3ʹ end of 13 UVs is a ribosome-binding, T-shaped structure (TSS) that was first characterized in β-carmovirus TCV (45–47). The TSS, composed of three hairpins and two pseudoknots, folds into a tRNA-like structure that binds to 60S ribosomal subunits and 80S ribosomes (Fig. 8A; Fig. S4. Group 2 ULVs do not have sequences that can fold into the full set of structures necessary to form a TSS (25). Of the Group 1 ULVs, only AgULV has the complete set of hairpins and pseudoknots in the same locations as in UVs (Fig. 8C). However, unlike every other known TSS, the two 5ʹ hairpins in the AgULV TSS are not juxtaposed, but rather are separated by an adenylate. The impact of this additional residue on the 3D structure of a TSS is not known. Finding a TSS in AgULV supports a close evolutionary relationship between this ULV and UVs.

### Most Group 1 ULVs contain a 3ʹ proximal, branched ISS 3ʹCITE not found in UVs or Group 2 ULVs

**Fig 9:**
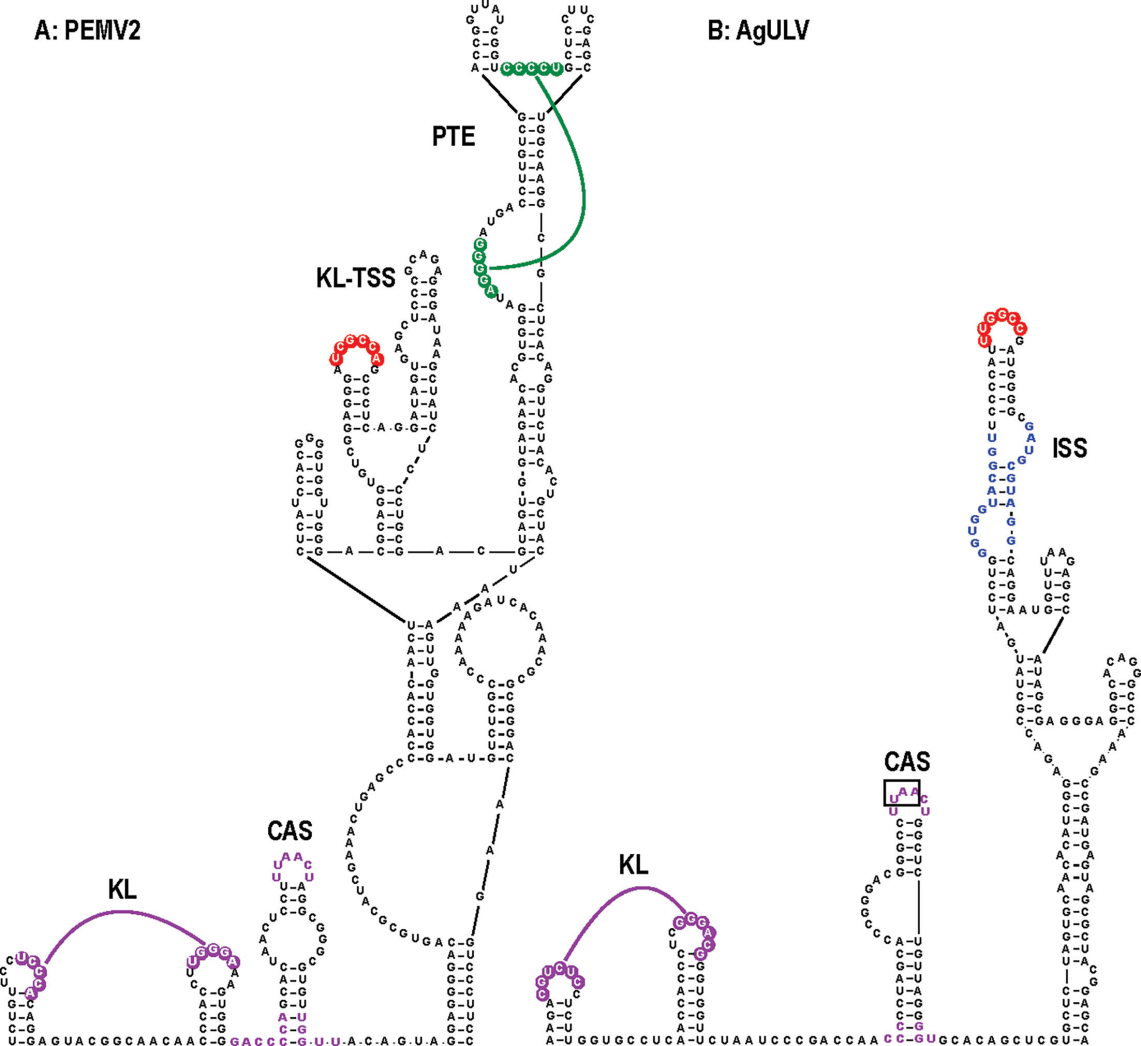
3ʹCITE region of PEMV2 and predicted 3ʹCITE region for AgULV. (**A**) 3ʹCITE region of PEMV2. The panicum mosaic virus translation enhancer (PTE) and kissing loop-T-shaped structure (KL-TSS) CITEs are shown. PTE 3ʹCITEs normally have their LDI sequence in the apical loop of the 5ʹ side hairpin, but in PEMV2, this function is transferred to the KL-TSS (in red). PEMV2 contains a typical CAS hairpin, and the conserved residues are in purple. Most CAS hairpins are preceded by two simple hairpins joined by a kissing loop (KL) interaction. CAS has different functions in translation depending on the virus (50) and is always located a short distance upstream of highly branched structures that contain known or putative 3ʹCITEs (Fig. S4). (**B**) Predicted structure in the 3ʹ UTR of AgULV includes a canonical CAS and typical KL hairpins just upstream of a branched structure containing an ISS 3ʹCITE. Boxed residues in the CAS mark the termination codon for ORF3. Conserved ISS sequences are in blue, and apical loop residues (in red) are complementary to the apical loop of a hairpin at the 5ʹ end of the putative sgRNA (Fig. S5A).

In UVs, the 3ʹ proximal TSS is only used for translation of the sgRNA (24, 49). For translation of the gRNA, UVs employ a second 3ʹCITE that is centrally located in the 3ʹ UTR. These 3ʹCITEs are positioned just downstream of a hairpin known as the CITE-associated structure (CAS) (50) (Fig. 9A; Fig. S4). All CAS hairpins have a symmetrical or asymmetrical internal loop and conserved sequences in the apical loop and base of the stem. Just upstream of the CAS in 13 UVs are a pair of simple hairpins connected by a kissing-loop (KL) interaction. UVs connect these 3ʹCITEs with 5ʹ end coding or non-coding sequences through an LDI (Table 1). In 5 UVs, the interacting sequences include “GCCA” and “UGGC,” which are also commonly found in carmovirus 3ʹCITE LDIs (43).

All UV CITEs downstream of CAS elements are positioned on scaffolds, many with multiple branches, suggesting that extensions may facilitate the LDI with the 5ʹ end (Fig. 9A; Fig. S4) (50). Nine UVs have one of two types of barley yellow dwarf translation enhancers (BTE-A or BTE-B) (8), and PEMV2 has a combination of a panicum mosaic virus translation enhancer (PTE) and a different version of a ribosome-binding TSS (Fig. 9A) (49). Five UVs have a similar, previously unreported structure with three hairpins atop an extended scaffold, with either the 5ʹ side or 3ʹ side hairpin containing the LDI sequence in their apical loop (Fig. S6). We have termed this 3ʹCITE an “umbravirus translation enhancer” (UTE).

We began our examination of Group 1 ULV 3ʹCITEs by searching for the presence of a CAS hairpin, which would be located just upstream from the 3ʹCITE. Two Group 1 ULVs, AgULV and ArULV, contain a canonical CAS hairpin along with the associated upstream KL hairpins (Fig. 9B; Fig. S5B). Just downstream of the CAS in AgULV is a CITE-like structure atop a branched scaffold. Sequence and structure analysis of the upper hairpin in the CITE-like structure revealed that it is a 3ʹCITE known as an I-shaped structure (ISS), previously found in γ-carmoviruses, aureusviruses, and tombusviruses, but not UVs (43). ISS are distinguished by two conserved sequences on opposing sides of the apical stem, which comprise portions of two bulged loops that are important for binding to eukaryotic translation initiation factor eIF4F (51). The LDI sequence was reported to be in the apical loop of ISS, which, for the putative AgULV ISS, is 5ʹ UUGGCCG (the commonly found UGGC LDI sequence is underlined). Although the reported AgULV gRNA sequence is missing its 5ʹ terminal region, which may include the LDI sequence, the AgULV sgRNA (identified as beginning with a CCS just upstream of the ORF3 initiation codon) contains a 5ʹ proximal hairpin with a fully complementary sequence (5ʹ GGCCAA) in its apical loop (Fig. S5A). Altogether, these features strongly suggest that AgULV, as with UVs, has two 3ʹCITEs: a TSS at the 3ʹ end and an upstream ISS associated with a CAS element.

We also examined sequences downstream of the ArULV CAS for the presence of a CITE-like structure (Fig. S5B). Curiously, the CAS was upstream of a short hairpin that did not resemble any known 3ʹCITE. No sequences near the annotated ORF3 and ORF4 start sites were complementary to the internal or apical loop of this structure. Thus, the ArULV 3ʹCITE remains to be determined.

**Fig 10:**
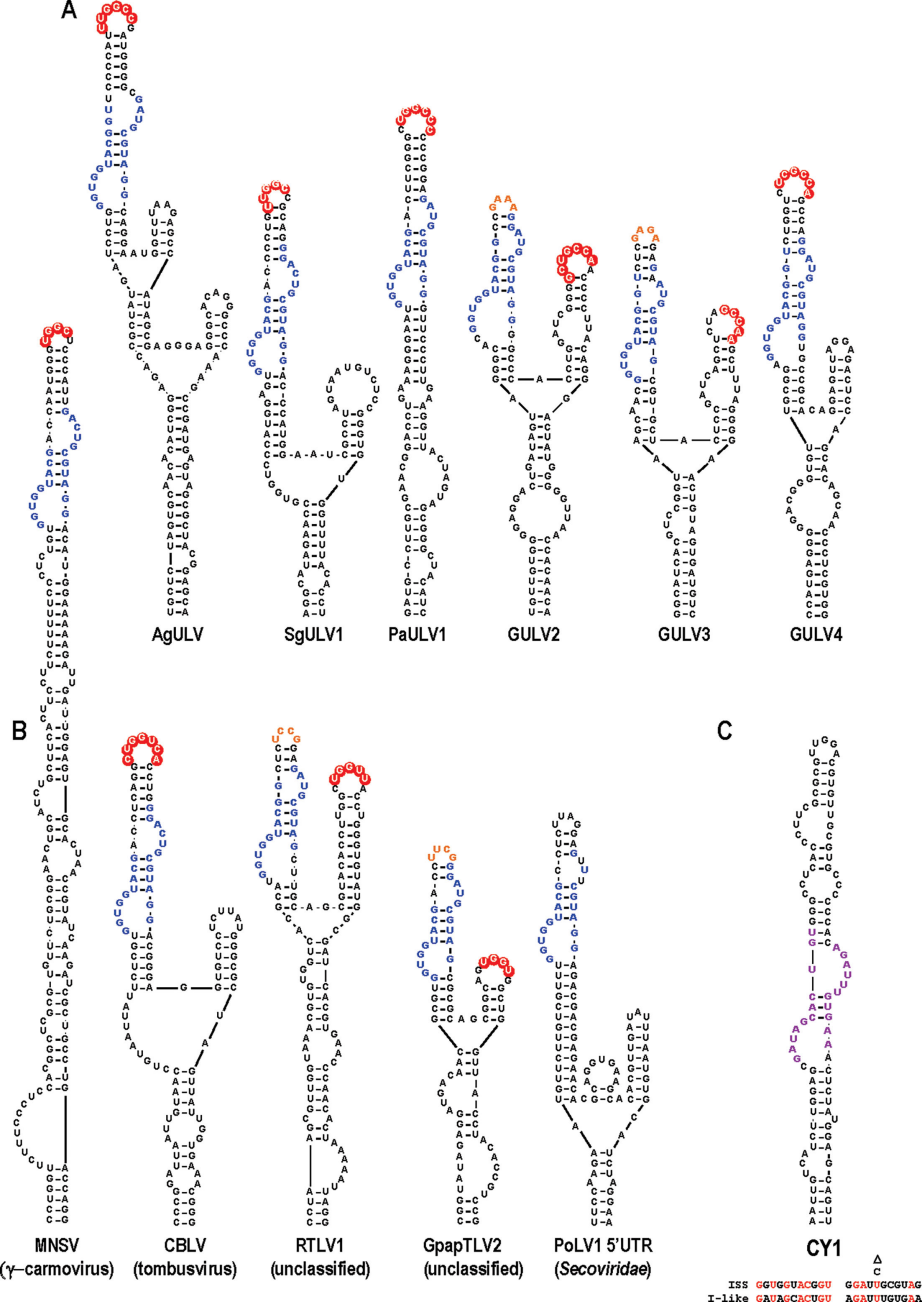
ISS 3ʹCITEs found in Group 1 ULVs and other plant viruses. (**A**) Compilation of ISS 3ʹCITEs from Group 1 ULVs. Conserved ISS sequences are in blue, and apical loop residues (in red) are complementary to the apical loop of a hairpin at the 5ʹ end of the gRNA and/or sgRNA (Fig. S5). GULV4 and GULV5 ISS have identical sequences, and only one is shown. Orange residues denote highly stable GNRA or UNCG tetraloops capping ISS stems that do not contain the LDI in the apical loop. (**B**) ISS in other viruses. Red shading denotes complementary sequences that could participate in the LDI with the 5ʹ end. Note that the PoLV1 ISS is located in the 5ʹ UTR of the gRNA. Virus abbreviations are defined in Table S1. (**C**) Similar 3ʹ CITE of Group2/Class2 CY1. Sequences that are conserved in all Group2/Class 2 ULVs are in purple. Similarity between this sequence and the ISS conserved sequence is shown below the structure.

Searches of the remaining Group 1 ULV sequences revealed that all have an ISS 3ʹCITE just upstream from hairpin H5 (Fig. 10A: note that since GULV4 and GULV5 have identical ISS sequences, only one is shown). The location of these ISS is identical to the positioning of various 3ʹCITEs just upstream of H5 in carmoviruses (52). Unlike previously identified ISS (see melon necrotic spot virus [MNSV] ISS, Fig. 10B), 6 of the 7 Group 1 ISS are branched structures rather than being “I-shaped.” Furthermore, two Group 1 ULVs (GULV2 and GULV3) have the LDI sequence in the apical loop of a second hairpin, and not in the primary ISS hairpin that is capped by a highly stable GNRA tetraloop. This suggests that the binding location of the 43S translation initiation complex can be separate from the LDI.

To determine if this branched ISS configuration is specific to Group 1 ULVs, we searched other plant viruses for the ISS conserved sequences and formation of the conserved bulged loop structure. Similar ISS were predicted for some additional members of the *Tombusviridae*, including cucumber Bulgarian latent virus (CBLV), rice tombus-like virus 1 (RTLV1), and Guiyang Paspalum paspaloides tombus-like virus 2 (GpapTLV2) (Fig. 10B). These ISS all form branched structures, and two (RTLV1 and GpapTLV2) have a sequence capable of pairing with their gRNA 5ʹ ends in a separate hairpin. Interestingly, one member of the *Secoviridae* family (Poaceae liege virus 1) contained an apparent ISS in its 5ʹ UTR (Fig. 10B). Although CITEs are known to function at the 5ʹ end of an mRNA (53, 54), to our knowledge, this is the first ISS in a 5ʹ location. Interestingly, Group 2/Class 2 ULVs have a similarly shaped 3ʹCITE denoted as “ISS-like” (44) and no discernible CAS-like hairpin (Fig. 10C). The ISS-like and ISS 3ʹCITEs share 45% identical residues in their conserved segments (Fig. 10C, bottom); however, the ISS-like 3ʹCITE binds to eIF4G and does not contain a discernible LDI with the gRNA 5ʹ end (44). Although UVs have not been identified with ISS 3ʹCITEs, finding that the majority of Group 1 ULVs have ISS, including closely related AgULV, suggests that there exist undiscovered UVs with a similar 3ʹCITE.

### Conclusion

Building on the model in Fig. 5, ULVs likely evolved from an ISS-containing UV by first generating an AgULV-like virus from the loss of the embedded UV ORF3. This was followed by the duplication of the 30K MP ORF4 and divergent evolution of paralogs into a capsid-like protein and a protein of currently unknown function, neither of which is required for systemic infection of Group 1 GULV4. Since UV ORF3 encodes an essential, multifunctional protein that allows transit through the phloem and confers protection from nonsense-mediated decay (10), these functions must have been transferred to either a helper virus and/or by co-opting a host RNA movement system. Studying the differences between UV and AgULV infection in the presence and absence of their helper viruses should provide insights into how UV ORF3 became dispensable. Importantly, this model posits that jelly roll capsid-like proteins can be derived from 30K MPs and not simply the reverse, as was previously suggested (11). Evolution of Group 1 ULVs from an AgULV-like ancestor eliminated the 3ʹ terminal TSS and the CAS and positioned the remaining ISS 3ʹCITE closer to the 3ʹ end, similar to most carmoviruses (52). Evolution of Group 2 viruses from Group 1 viruses included a substantial redesign of the frameshifting site. We previously speculated that the complex frameshifting site of Group 2 viruses is necessary to achieve high levels of recoding found for CY1 *in vitro* (~29% compared with ~5% for UVs) (14), but why only Group 2 ULVs would require such high RdRp levels remains unknown.

An interesting question is what benefits drove this evolutionary process? The existence of ULVs demonstrates that viral MPs are dispensable, but the vast majority of plant viruses encode their own MPs. This suggests that, although plant viruses can use the host RNA movement system, the inability to regulate this endogenous system selects for maintenance of viral-encoded MPs. Our previous findings suggest that ULVs benefit from using host MPs by gaining the ability to infect a wider host range (15), since movement would not be restricted by incompatibility. between encoded MPs and host docking proteins associated with plasmodesmata, the conduits through which viruses or viral RNAs must transit (35). Since ULV infections are mostly asymptomatic in their natural hosts, it is tempting to speculate that the dependence on host MPs might lessen deleterious virus-host interactions. Additionally, by evolving a capsid-like protein from an encoded MP, capsid-forming ULVs gained independence from their host-restricted helper viruses and potentially further increased their host range. Continued research on ULVs will undoubtedly further our understanding of plus-strand RNA plant viruses and the selective trade-offs driving their evolution.

## MATERIALS AND METHODS

### Phylogenetic analysis

To evaluate the relationship between UV and ULV RdRp sequences, the annotated amino acid sequences were aligned with MAFFT (version 7.526) using iterative refinement and global alignment parameters (G-INS-i). The resulting alignment is found in Data set S1. For the comparison of ORF3 and ORF4 sequences, the respective sequences were first evaluated as to whether they were preceded by a CSS. For sequences that lacked an upstream CSS but contained a downstream methionine that did contain a nearby upstream CSS, the sequence beginning at the downstream methionine was used. Alternatively, for Group 2/Class 3 ORF4 sequences predicted to be translated by a readthrough of the ORF3 stop codon (WULV, GULV1), ORF3 and ORF4 sequences were combined as a single ORF3. This alignment can be found in Data set S2. For the nucleotide alignment of ORF3 and ORF4, the annotated sequences were used. Maximum-likelihood phylogenetic trees for the amino acid alignments were generated using raxmlHPC (version 8.2.12) with optimized substitution and gamma distribution rates (PROTGAMMAAUTO). For the nucleotide alignments, a general time-reversible model with gamma distribution rates and invariable frequency was used (GTRGAMMAI). Trees were generated using the ggtree package (version 4.0.1) in R (version 4.5.1). The following accession numbers were used: OPMV (EU151723), TBTV (MZ404117), PaeUV (OP053684), ETBTV (MW113249), GRV (OL999579), CjUV (KX883095), RCUV (MG596234), PEMV2 (U03563), PicUV1 (OL472231), IxYMoV2 (NC_034243), PMMoV (MH922775), CMoV (PP766558), WCMoV (LT615232), PasUV (PP766564), AgULV (OP660856), ArULV (OQ102001), PaULV (OM514399), PMeV2 (KT921785), PpVQ (MT113180), BabVQ (MT113182), WULV (OK573479), GULV1 (OP886321), PlULV (OL330774), SbaVA (MK211274), PULV (OM419177), CY2 (MT893741), CY1 (JX101610), OULV (MH579715), TULV (OK018180), MULV (OM937759), JgULV (OM937760), EmaV (MF415880), SULV (MN868593), SgULV1 (PP996017), SgULV2 (PP996018), FULV (MW480892), GULV2 (OR947505), GULV3 (OR947506), GULV4 (OR947507), GULV5 (PQ893052), WSVA (OP584700), CUV2 (OP886452), CMoMV (PP766559), TMV (NP_597748), and BYDV (NC_002160).

### Protein structure prediction

Tertiary and higher-order structures of ORF3- and ORF4-encoded proteins from UVs and ULVs, along with the TMV 30K MP and the BYDV CP, were modeled using the Alphafold3 web server and imaged using PyMol (Version 3.1.0). All predicted structures can be found at Figshare DOI: https://doi.org/10.6084/m9.figshare.31281964. Cartoon structures were colored either by b-factor to indicate model confidence or based on predicted secondary structural elements. Surface structures shown for the higher-order structures were colored based on the predicted electrostatic potential as predicted by the APBS Electrostatics plugin using PDB2PQR.

### RNA structure predictions

Full-length viral RNA structures were predicted using Linearfold (55), and local RNA regions were also folded using RNAStructure (https://rna.urmc.rochester.edu/RNAstructureWeb/Servers/Predict1/Predict1.html). RNA structures were viewed using RNAcanvas (56) and modified by hand using RNAcanvas pairing functions to conform to known structures based on the presence of conserved sequences. The “Motif Search” command within RNAcanvas was used to search for Watson-Crick LDIs with FSE and 3ʹCITE interactions with 5′ sequences.

### Plasmid constructs

Plasmid pCB301-GULV4 containing the full-length reported sequence of GULV4 downstream of a CaMV 35S promoter and upstream of a hepatitis delta virus ribozyme was kindly provided by Silvec Biologics, Inc. The desired mutations were introduced using custom-designed oligonucleotide primers (Integrated DNA Technologies). The resulting PCR products were subjected to DpnI digestion before transformation into competent DH5α *E. coli* cells. Sanger sequencing (Poochon Scientific) for the detection of desired mutations was performed on PCR products synthesized using primers flanking the mutation site, 5’- ATGAGCAACAATACCACTGTGAGAGTGC and 5’- CTAGAGCAGGTAGCCTCCGTTCTG for ORF3 and 5’- ATGTTTTCACCGTTTTTGAGCGTAGC and 5’- CTACTTTTCGCCTCCACACGC for ORF4.

### Vacuum infiltration of *N. benthamiana*

Electrocompetent *A. tumefaciens* strain GV3101 was transformed with binary vectors using electro cell manipulator ECM 395 (BTX) and then plated on LB with antibiotics (20 mg/L rifampicin and 50 mg/L kanamycin) for 72 h at 28°C. A single colony was transferred to 3 mL of Luria broth (LB) supplemented with antibiotics and incubated overnight with shaking at 28°C. The seed culture was transferred to 150 mL of fresh LB with antibiotics until an OD_600_ of 0.8 was reached. The culture was centrifuged at 4,000 × *g* for 20 min. Pelleted cells were resuspended in infiltration MA buffer (10 mM MgCl_2_, 100 μm acetosyringone) and incubated at room temperature for 2 h prior to infiltration. After incubation, the final concentration was adjusted to an OD_600_ of 0.4 for viral expression constructs and an OD_600_ of 0.2 for the silencing suppressor construct expressing TBSV p19. *N. benthamiana* at the 8-true leaf stage was immersed upside down in Agrobacterium-infiltration solution when applying the vacuum (0.8–0.9 bar) for 3–5 s.

### Virus detection

Tissue from *N. benthamiana* (100 mg) was collected from individual plants, and RNA extraction was performed using Trizol (Invitrogen) following the manufacturer’s protocol. Total RNA (~200 ng) was used to synthesize cDNA using M-MuLV reverse transcriptase (New England Biolabs) and random hexamer primers. PCR (25 cycles) was conducted using GULV4-specific primers 5′- GGCAATTATCCATGCTCACCC and 5′- CGTAATCTCCGACCCACTGTC, amplifying positions 8–153 for detection. Amplicons were visualized on a 1% agarose gel. Sanger sequencing, to verify maintenance of desired mutations, was performed on amplicons synthesized from primers described for plasmid construction verification.

### Poly(A) tailing of RNA and direct RNA nanopore sequencing

Approximately 500 ng of RNA was mixed with ddH_2_O to a volume of 15.5 μL. Two microliters of 10× buffer, 2 μL of 10 mM ATP, and 0.5 μL of *Escherichia coli* poly(A) polymerase (New England Biolabs, enzyme and buffer) were then added. Reactions were incubated at 37°C for 10 min and then terminated by the addition of 5 μL of 50 mM EDTA. Poly(A) tailed RNA was purified using RNAClean XP beads (Beckman Coulter) following the manufacturer’s instructions and resuspended in 12–16 μL of ddH_2_O. Sequencing libraries were prepared from poly(A)-tailed RNA samples using the direct RNA sequencing kit (SQK-RNA002) following the manufacturer’s instructions, including the synthesis of cDNA:RNA hybrids using oligodT (Integrated DNA Technologies) and SuperScript III (Invitrogen). Sequencing runs (18 h) were performed using version R9.4.1 flow cells and a MinION Mk1B device. The MinKNOW desktop application (Oxford Nanopore) was used for base calling of nanopore sequencing reads using the standard quality score threshold of seven for direct RNA sequencing (corresponding to at least 80% read accuracy).

## Data Availability

Predicted structures, as well as the structural predictions of higher-order complexes, have been included at Figshare DOI: https://doi.org/10.6084/m9.figshare.31281964.

## SUPPLEMENTAL MATERIAL

Data Set S1 jvi.02209-25-s0001.txt Multiple alignment data set used for Fig. 1D.

Data Set S2 jvi.02209-25-s0002.txt 3D structure prediction files in PDB format for Fig. 3.

Supplemental material jvi.02209-25-s0003.pdf Table S1; Fig. S1-S6.

ASM does not own the copyrights to Supplemental Material that may be linked to, or accessed through, an article. The authors have granted ASM a non-exclusive, world-wide license to publish the Supplemental Material files. Please contact the corresponding author directly for reuse.
