# Supplementary material for: Structural and phylogenetic analyses of umbravirus and umbra-like virus genomes suggest evolution of capsid-like proteins from 30K movement proteins
Source: J Virol. 2026 Mar 19;100(4):e02209-25. doi: 10.1128/jvi.02209-25 (PMC13098196; doi:10.1128/jvi.02209-25)
Supplement: Supplemental material — Table S1; Fig. S1-S6. [file jvi.02209-25-s0003.pdf]

Supplemental Table 1. Virus and other abbreviations		
**Viruses**	**Names**	**Accession numbers**
**Umbraviruses (order from Fig. 1D)**		
CMoV	Carrot mottle virus	PP766558
PasUV	Pastinaca umbravirus	PP766564
WCaUV	Wild carrot mottle virus	LT615232
CUV	Carrot umbravirus	OP886452
CMoMV	Carrot mottle mimic virus	PP766559
IxYMoV2	Ixeridium yellow mottle virus 2	NC_034243
PicUV1	Picris umbravirus 1	OL472231
PMMoV	Patrina mild mottle virus	MH922775
WSVA	White snakeroot virus A	OP584700
PEMV2	Pea enation mosaic virus 2	U03563
RCUV	Red clover umbravirus	MG596234
CjUV	Changjiang tombus-like virus 3	KX883095
GRV	Groundnut rosette virus	OL999579.1
ETBTV	Ethiopian tobacco bushy top virus	MW113249
PaeUV	Paederia scandens chlorosis yellow umbravirus	OP053684
OPMV	Opium poppy mosaic virus	EU151723
TBTV	Tobacco bushy top virus	MZ404117
**Group 1 Umbra-like viruses**		
GULV2	Grapevine umbra-like virus 2	OR947505
GULV3	Grapevine umbra-like virus 3	OR947506
GULV4	Grapevine umbra-like virus 4	OR947507
GULV5	Grapevine umbra-like virus 5	PQ89052
SgULV1	Switchgrass umbra-like virus 1	PP996017
PaULV1	Guiyang Paspalum paspaloides tombus-like virus 1	OM14399
ArULV	Arborvitae umbra-like virus	OQ102001
AgULV	Ageratum umbra-like virus	OP660856
**Group 2 Umbra-like viruses**		
SULV	Sugarcane umbra-like virus	MN868593
EMaV	Ethiopian maize-associated virus	MF415880
JgULV	Johnson grass umbra-like virus	OM937760
MULV	Maize umbra-like virus	OM937759
TULV	Teosinte umbra-like virus	OK18180
CY2	Citrus yellow vein associated virus-Delta	MT893741
CY1	Citrus yellow vein associated virus	JX101610
FULV	Fig umbra-like virus	MW480892
OULV	Opuntia umbra-like virus	MH579715
PULV	Parsley umbra-like virus	OM419177
GULV1	Grapevine umbra-like virus 1	OP886321
WULV	Wheat umbra-like virus	OK573479
PlULV	Poaceae liege umbra-like virus	OL330774
SgULV2	Switchgrass umbra-like virus 2	PP996018
SbaVA	Strawberry associated virus A	MK211274

PpVQ	Papaya virus Q	MT113180
BabVQ	Babaco virus Q	MT113182
PMeV2	Papaya meleira virus 2	KT921785
**Other viruses**		
CBLV	Cucumber Bulgarian latent virus	OL311696
GaMV	Galinsoga mosaic virus	NC_001818
GpapTLV2	Guiyang tombus-like virus 2	OM514400
MNSV	Melon necrotic spot virus	PV741264.1
PoLV1	Poaceae Liege virus 1	NC_076923
RTLV1	Rice tombus-like virus 1	MT317153
SCV	Saguoro cactus virus	SCU72332
TBSV	Tomato bushy stunt virus	NC_001554
TCV	Turnip crinkle virus	AY312063
TMV	Tobacco mosaic virus	NP_597748

**Additional Abbreviations**	
BTE	Barley yellow dwarf virus translation enhancer
CA	CA-rich sequence at frameshift site
CAS	CITE-associated structure
CCS	Carmovirus consensus sequence
CITE	Cap-independent translation enhancer
FSE	Frameshift stimulatory element
gRNA	Viral genomic RNA
HA	Hairpin upstream of FSE
HB	Hairpin downstream of FSE
HC	Hairpin downstream of HB
H5	Hairpin H5 involved in Ψ1
ISS	I-shaped structure CITE
KL	Kissing loop
LDI	Long-distance interaction
Pr	3’ terminal hairpin in UVs and carmoviruses
PRF	Programmed ribosomal frameshifting
PTE	Panicum mosaic virus translation enhancer
sgRNA	viral subgenomic RNA
TSS	T-shaped structure CITE
UTE	Umbravirus translation enhancer

Supplemental Figure Legends

SFig 1 ULV ORFs encoding full jelly-roll proteins contain a D-motif. (A) AlphaFold3 predictions of the CY2 ORF3 full-jelly roll capsid-like protein and PEMV2 ORF4 30K MP. Beta-strands are colored to indicate their numbering and the aspartic acid residue (the D motif) between the E and F strands is colored in cyan. (B) Amino acid alignment of ULV full jelly-roll protein sequences at the position of the potential D-motif in the CY2 capsid-like protein. (C) Amino acid alignment of AgULV ORF3 and GULV4 ORF4 full jelly-roll proteins (arrows) at the position of the known D-motif in UV 30K MPs.

SFig 2 Phylogenetic analysis of UV and ULV ORF3/4 nucleotide sequences. ORF3 and ORF4 (if present) nucleotide sequences from selected UVs and ULVs were aligned. From this alignment, a maximum likelihood phylogenetic tree was generated with scale bar representing nucleotide substitution rate per base. Note that since the ORF3 and ORF4 sequences from UVs and Group 2/Class 2 monocot ULVs overlap substantially, these sequences cluster together with no predicted substitution rates between them (top and bottom of the tree). For open-reading frames that overlap, the overlapping partner sequence is denoted in parentheses. The branches from specific Group 1 ULVs encoding both ORF3 and ORF4 are colored alike and the predicted protein structure is indicated by a green square (full jelly-roll), orange circle (partial jelly-roll), or blue circle (disordered).

SFig 3 GULV4 infects *N. benthamiana* systemically in the absence of the ORF3- or ORF4-encoded protein. Since GULV4 does not encode a silencing suppressor (Mikkelsen and Simon, unpublished) similar to umbraviruses (1), systemic infection following agroinfiltration is routinely performed with an added silencing suppressor, such as tomato bushy stunt silencing suppressor p19 (2). (A) Left, healthy plant; middle, plant vacuum-infiltrated with p19 only; right, plant vacuum-infiltrated with p19 and GULV4 WT. Pictures were taken at 2-weeks post-infiltration (wpi). Note that GULV WT infection causes necrosis of local (infiltrated) leaves. (B) RT-PCR results from systemic leaves of *N. benthamiana* infected with GULV4 WT or GULV4 with premature stop codons in ORF3 (ORF3-stop) or ORF4 (ORF4-stop) at 2-wpi. All PCR-positive plants displayed necrosis of local infiltrated leaves. (C) Sanger sequencing chromatographs of total RNA isolated from ORF3-stop or ORF4-stop plants at 2-wpi. Batch sequencing showed no evidence of reversion.

SFig 4 Known or predicted RNA structures for elements involved in UV translation and replication. Structures shown (from top to bottom) are for the 5' end of the reported gRNAs (if the long-distance interaction [LDI] sequence with the 3' cap-independent translation enhancer [CITE] is present), the CITE-associated structure (CAS) and 3'CITE, the 3' terminus, and the frameshifting site. Carmovirus consensus sequence (CCS) at the 5' ends of gRNAs are in green; Truncations at the ends of the reported sequences (see text and Fig.

2) are denoted by [T]. ORF1 start codon (if present) is shaded green. UTE, umbravirus translation element; BTE-A and BTE-B, barley yellow dwarf virus translation element. Residues shaded in red denote complementary bases putatively engaged in the LDI between the 3'CITE and 5' sequences; conserved CAS sequences are in purple; orange shaded sequence upstream of the ORF1 stop codon (yellow) is the -1PRF slippery sequence. Gray-shaded sequences are complementary between the FSE and the 3' end; purple sequence in the frameshifting structure is the newly discovered CA sequence; see Figs. 7 and 8 legends for additional labeling of hairpins and pseudoknots. Viruses are shown in the same order as in Fig. 1D. (A) CMoV. (B) PasUV. (C) WCMoV. (D) CMoV. (E) CUV. (F) IxYMoV2. Note that the single available IxYMoV2 sequence is truncated at both 5' and 3' ends. Additional potential kissing loop (KL) hairpins are shown upstream of the CAS-associated KL hairpins. (G) PicUV. (H) PMMoV. (I) WSVA. The single available WSVA sequence is truncated at the 3' end. (J) PEMV2. (K) RCUV. The 3'CITE in RCUV is unique among the UVs. (L) CjUV. (M) GRV. (N) ETBTV. (O) PaeUV. (P) OPMV. (Q) TBTV.

SFig 5 ULV predicted RNA structures for elements known to be involved in translation and replication of UVs. See legend SFig. 4 for details. (A) AgULV. Dots denote the ORF3 stop codon. Conserved CAS residues are colored purple. (B) ArULV. Conserved CAS residues are colored purple. (C) PaULV1. Note that PaULV1 did not contain an LDI with the 3' end. However, a 10 nt complementary Watson:Crick sequence was present between the FSE lower asymmetric loop and the apical loop of HA (dark gray). (D) SgULV1. (E) GULV2. (F) GULV3. (G) GULV4. (H) GULV5.

SFig 6 New UV 3'CITE. Predicted structures of the new 3'CITE denoted as the umbravirus translation element (UTE). These three hairpin "pitchfork" structures with the LDI sequence either in the apical loop of the first or third hairpins (in red) have not previously been reported.

Supplemental References

1. Taliansky ME, Robinson DJ. 2003. Molecular biology of umbraviruses: phantom warriors. J Gen Virol 84:1951-1960.

2. Qiu W, Park J, Scholthof H. 2002. Tombusvirus p19-mediated suppression of virus-induced gene silencing is controlled by genetic and dosage features that influence pathogenicity. Mol Plant Microbe Interact 15:269-280.

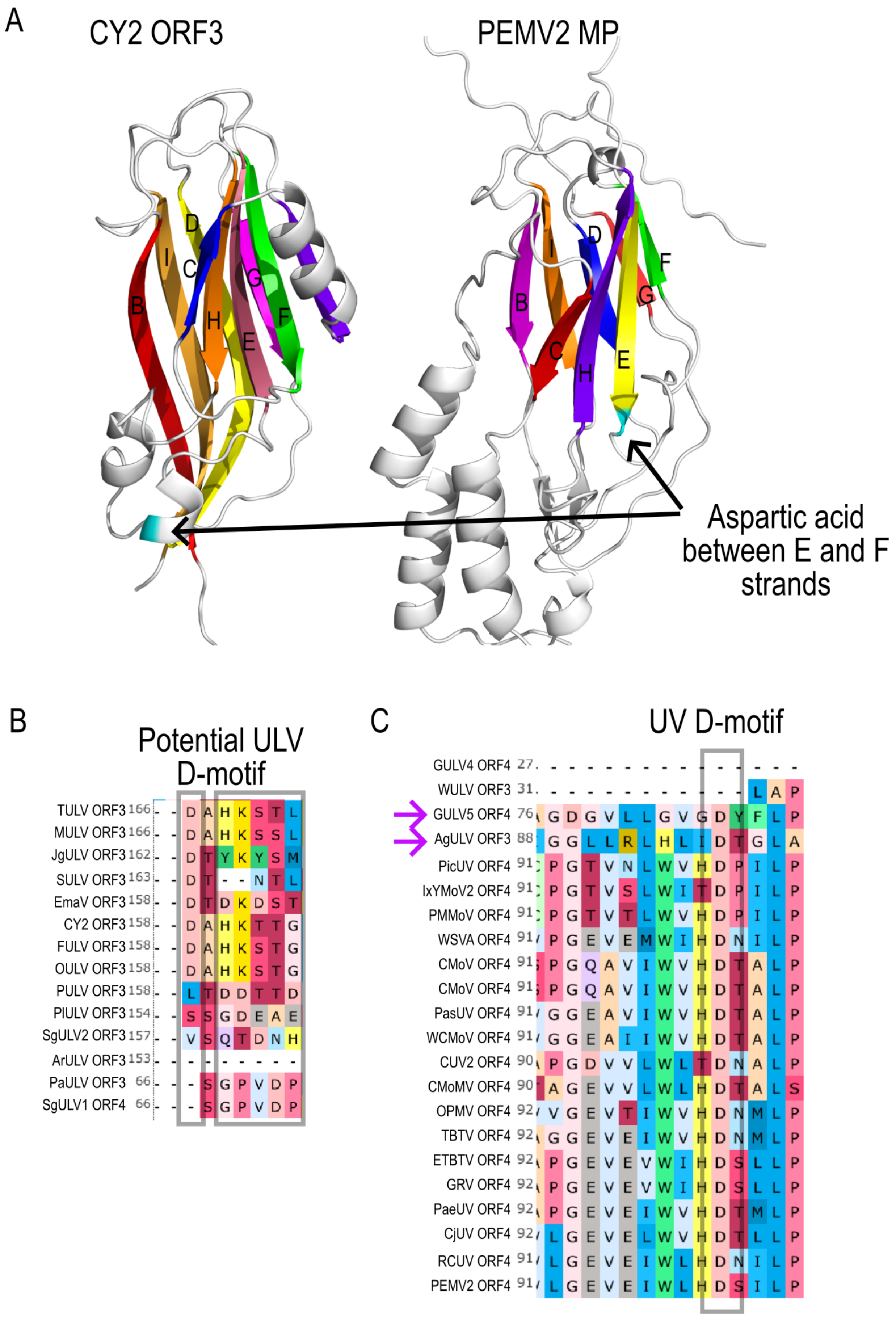

Supplemental Figure 1

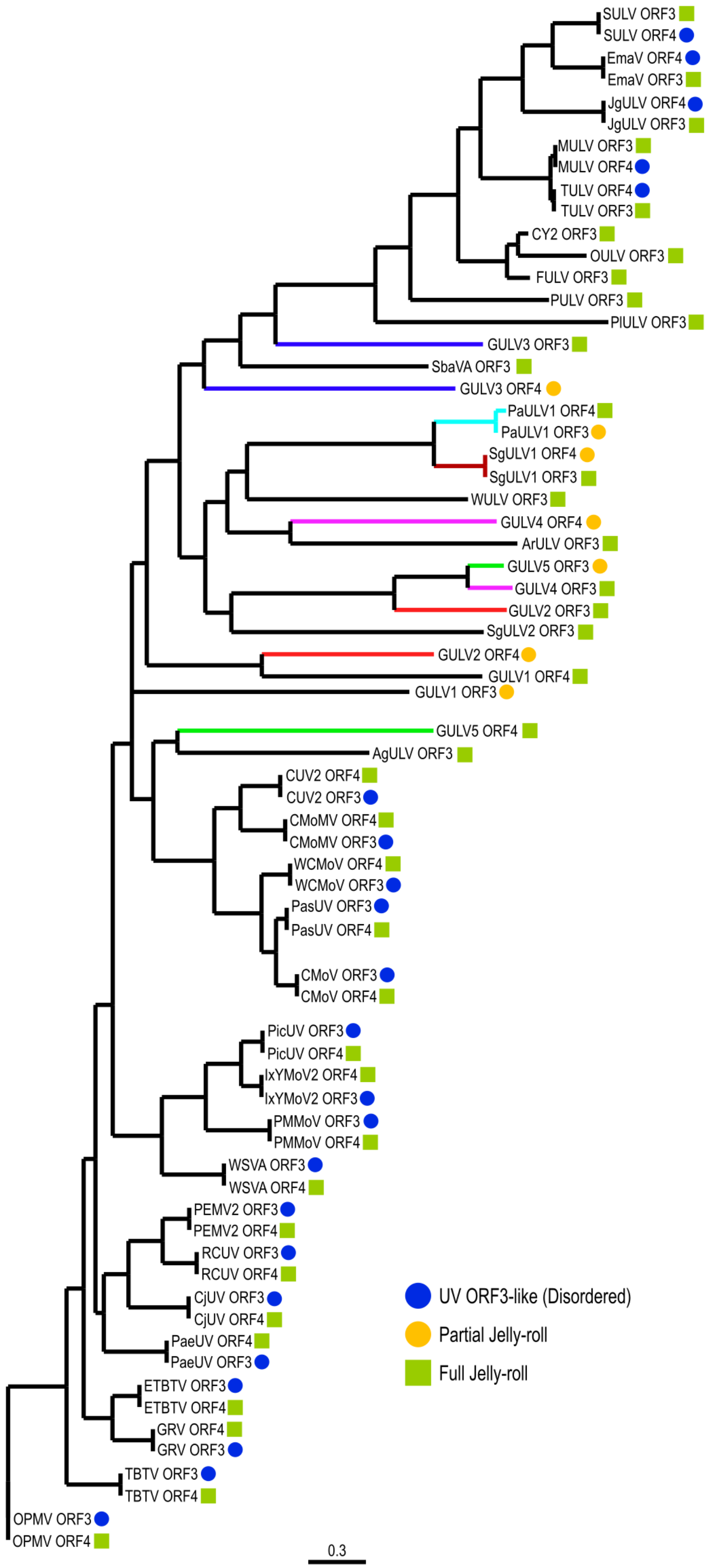

Supplemental Figure 2

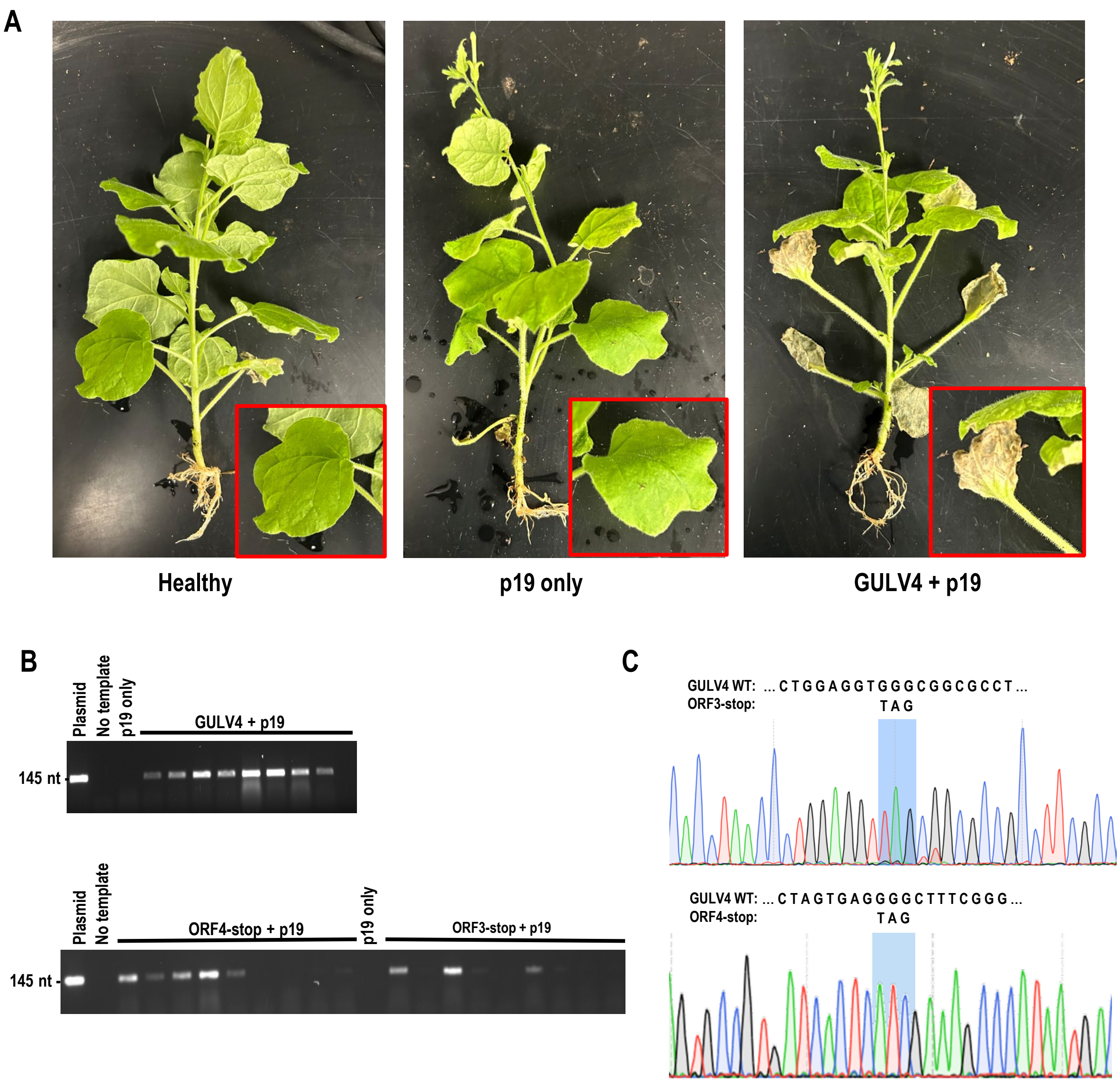

Supplemental Figure 3

A: CMoV

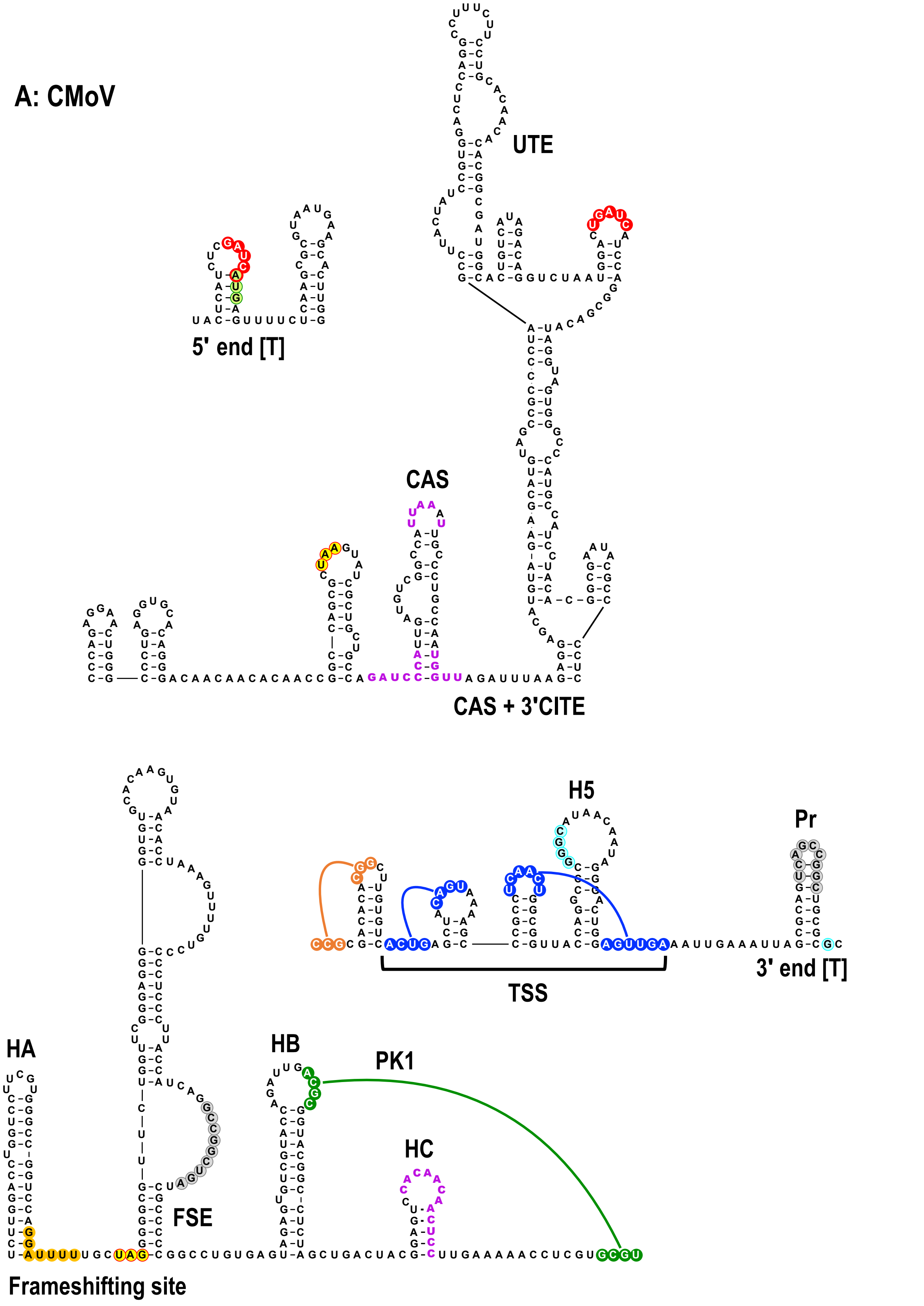

Supplemental Figure 4A

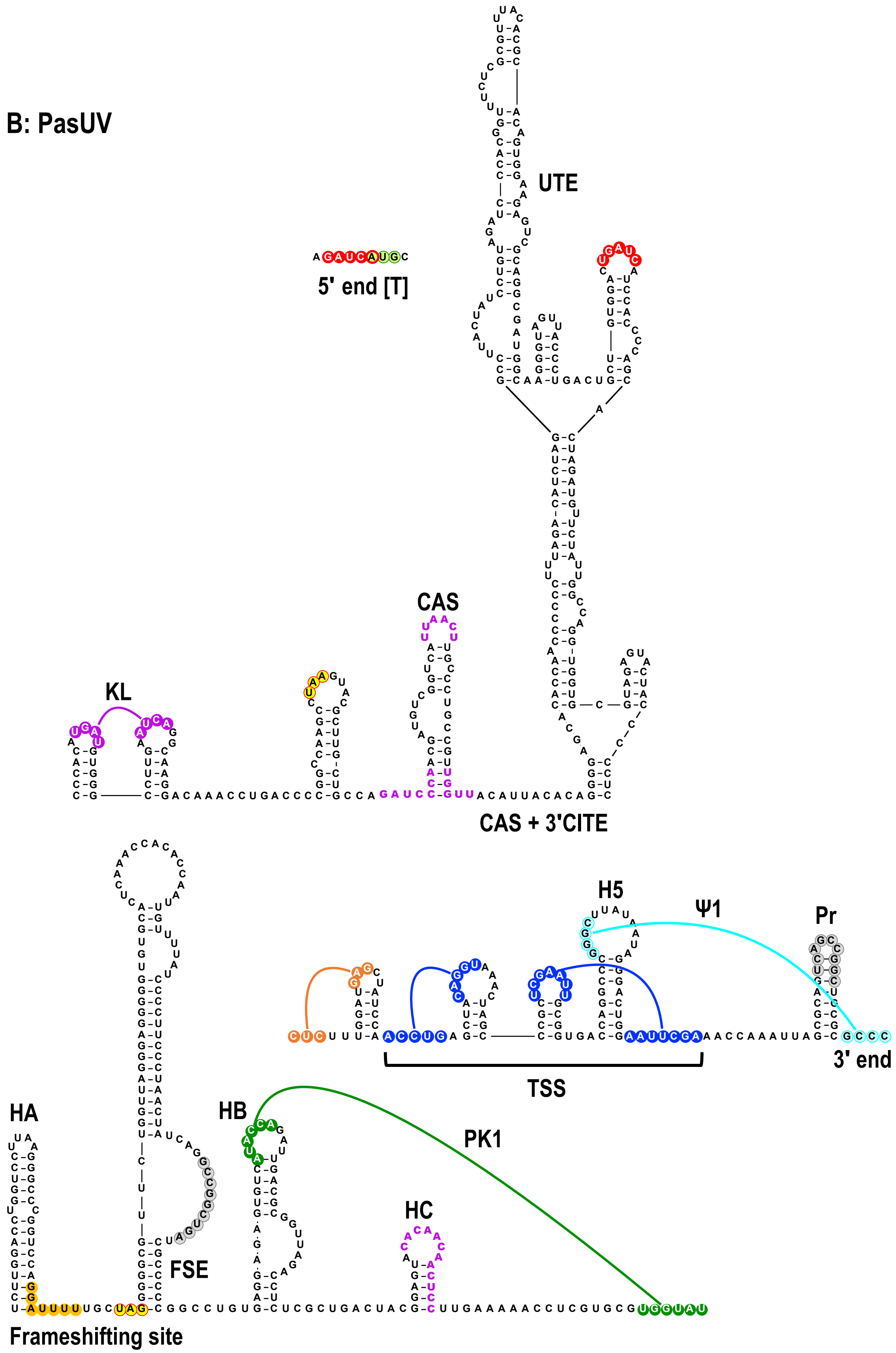

Supplemental Figure 4B

C: WCMoV

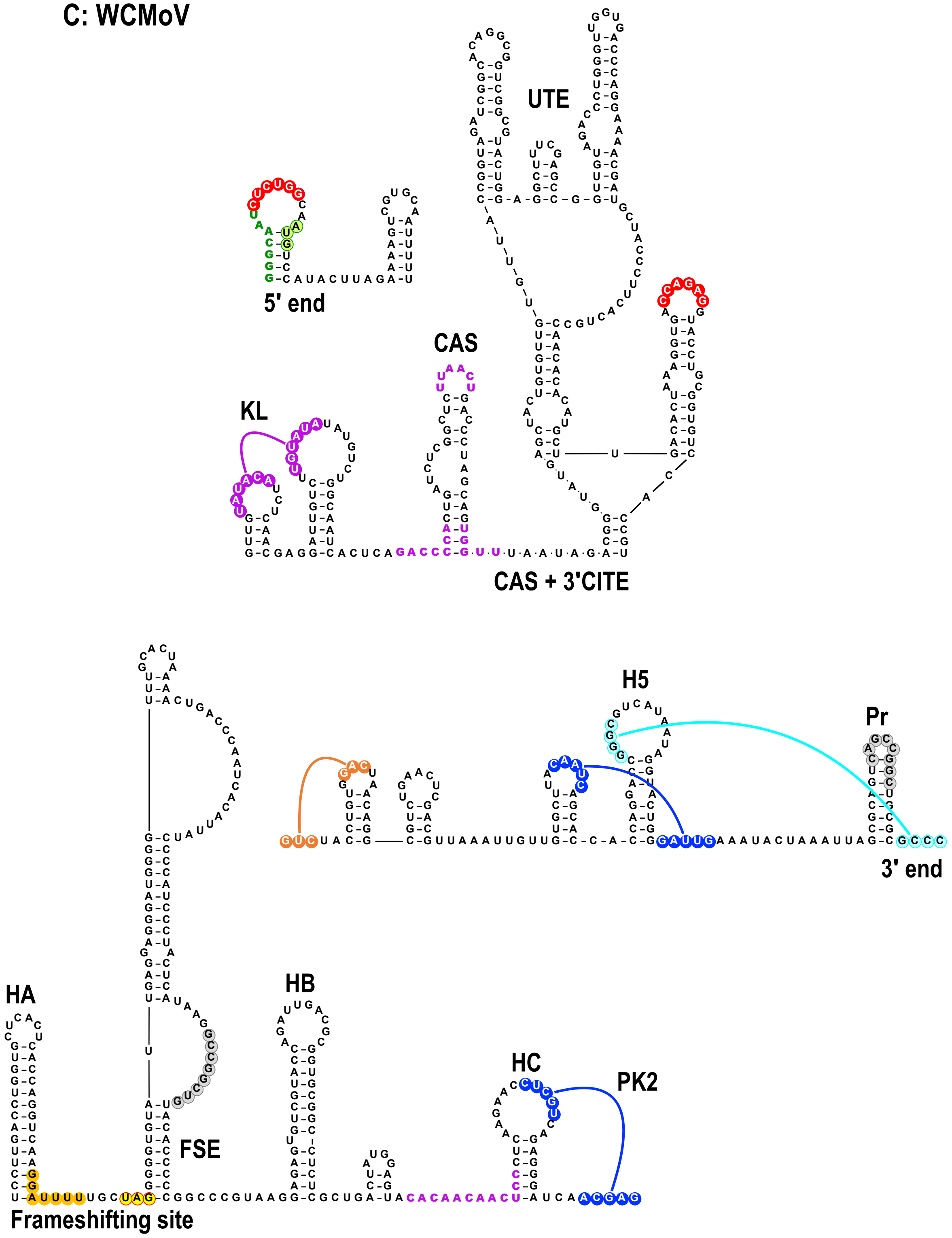

Supplemental Figure 4C

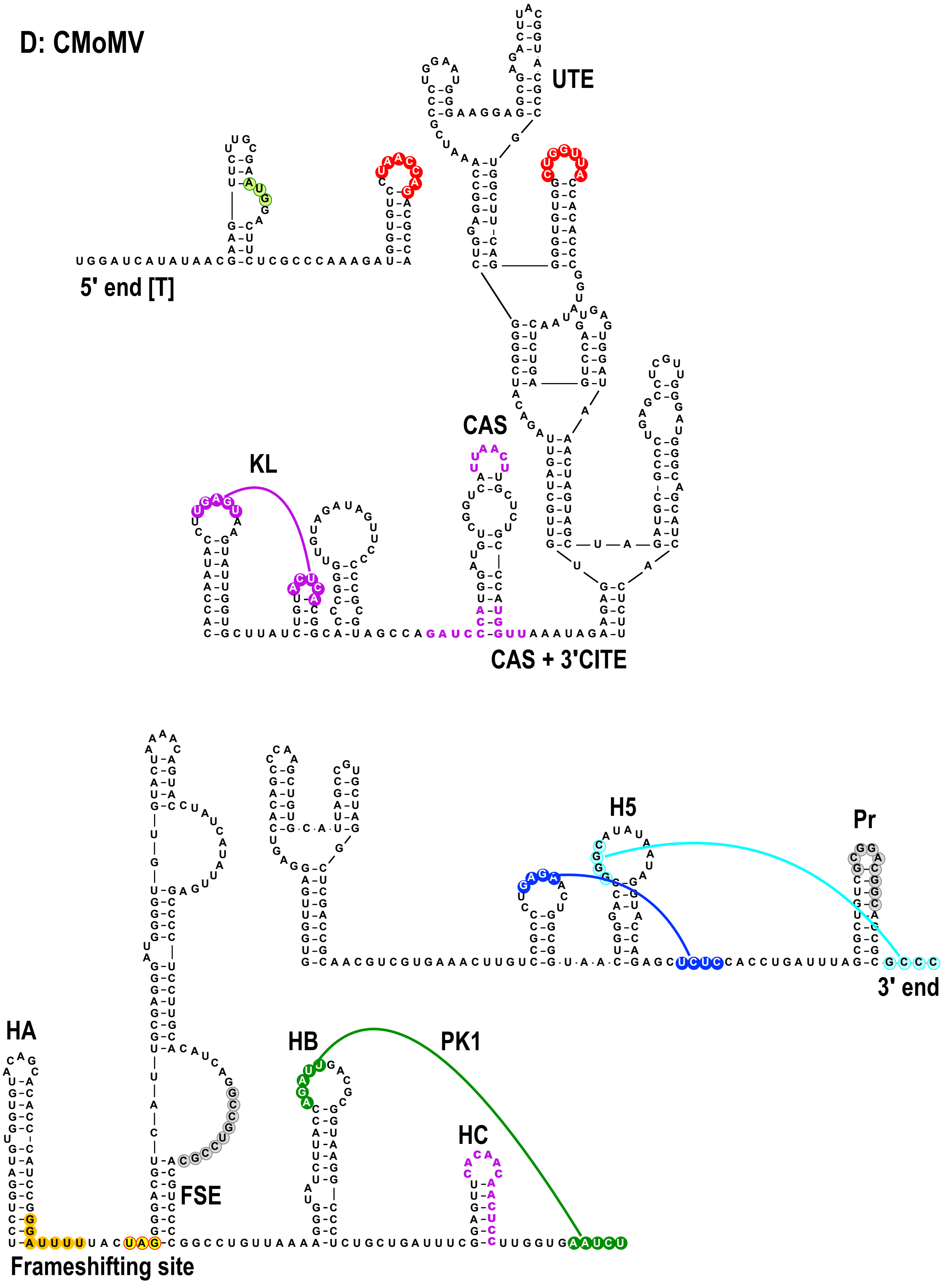

Supplemental Figure 4D

E: CUV

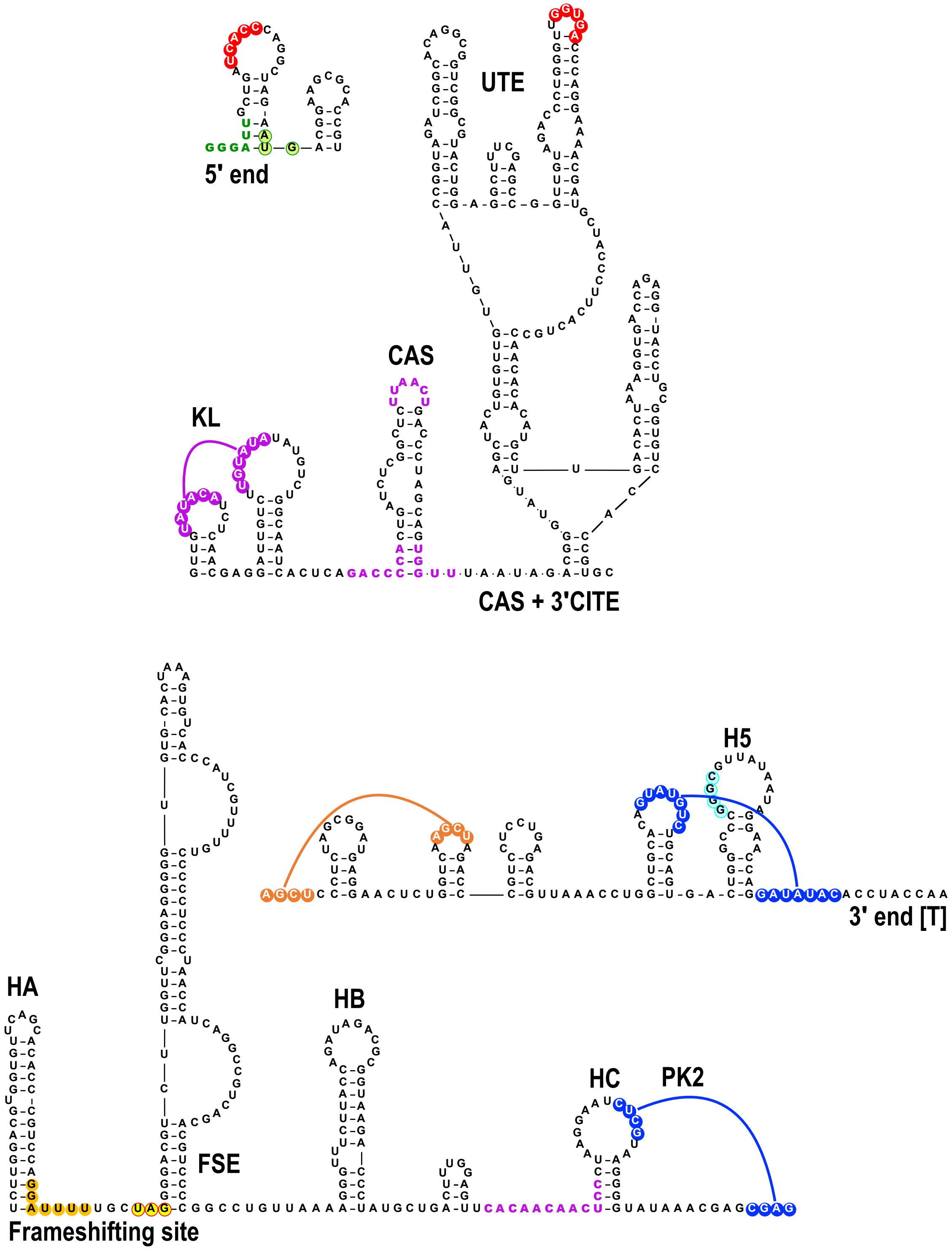

Supplemental Figure 4E

F: IxYMoV2

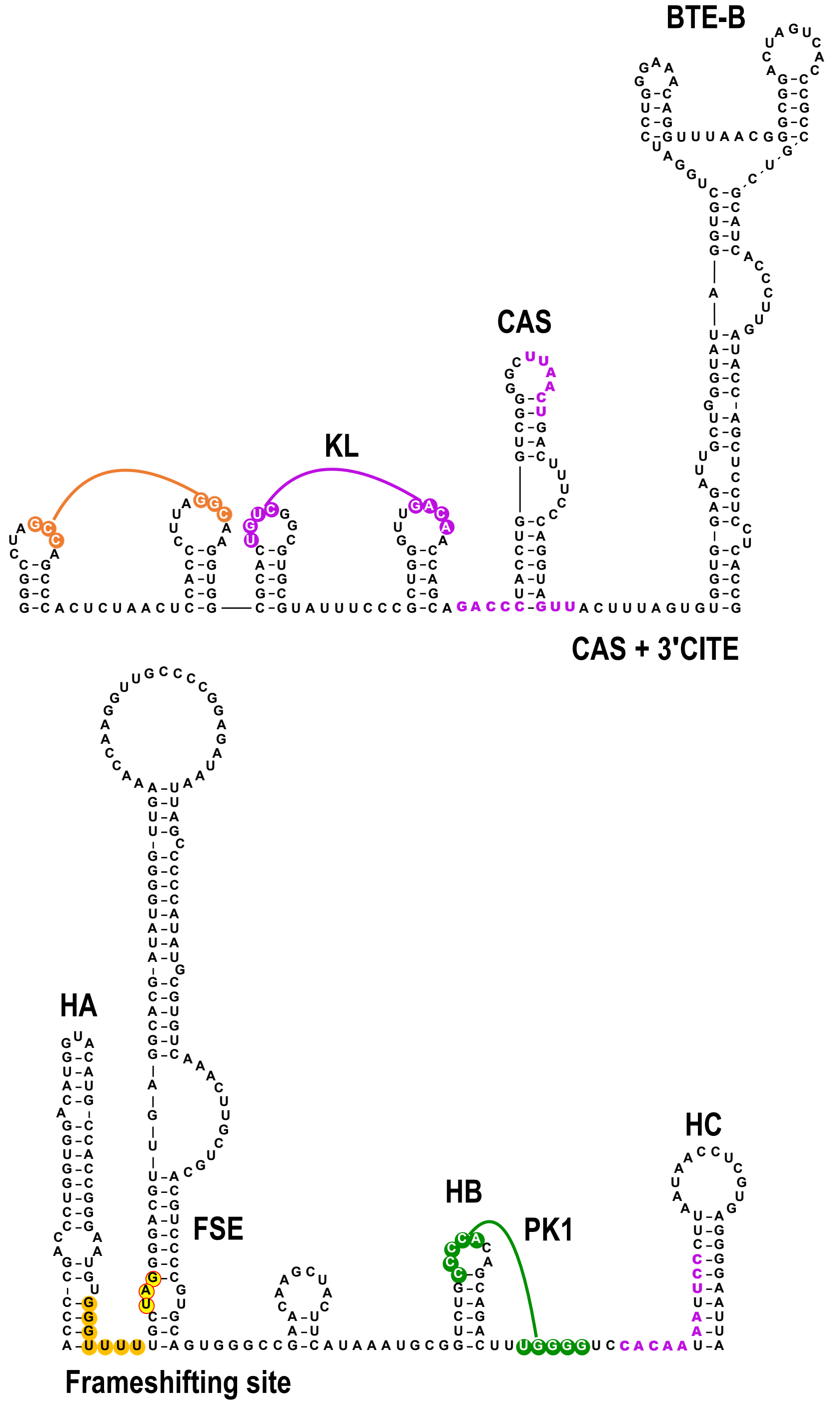

G: PicUV

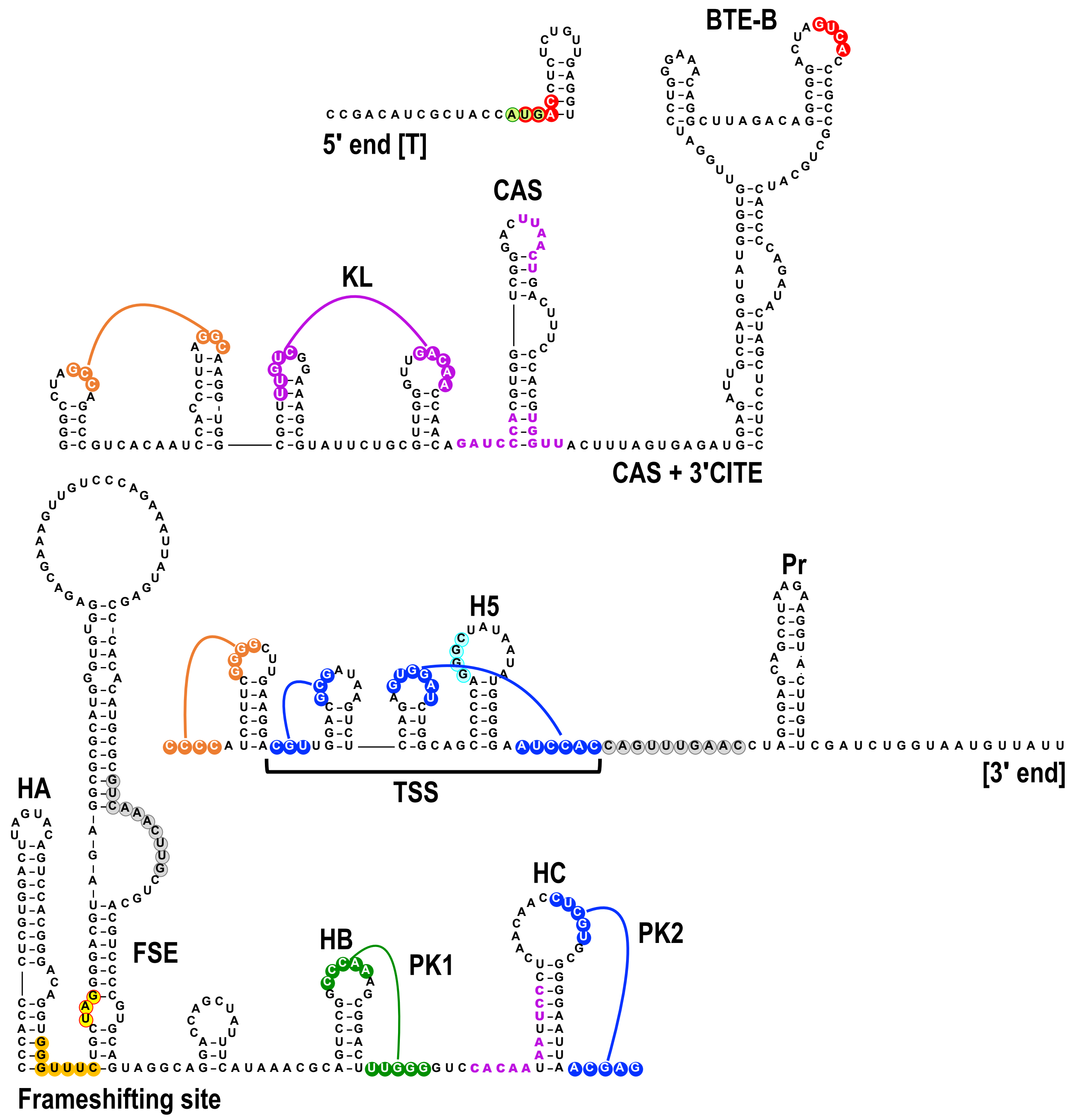

Supplemental Figure 4G

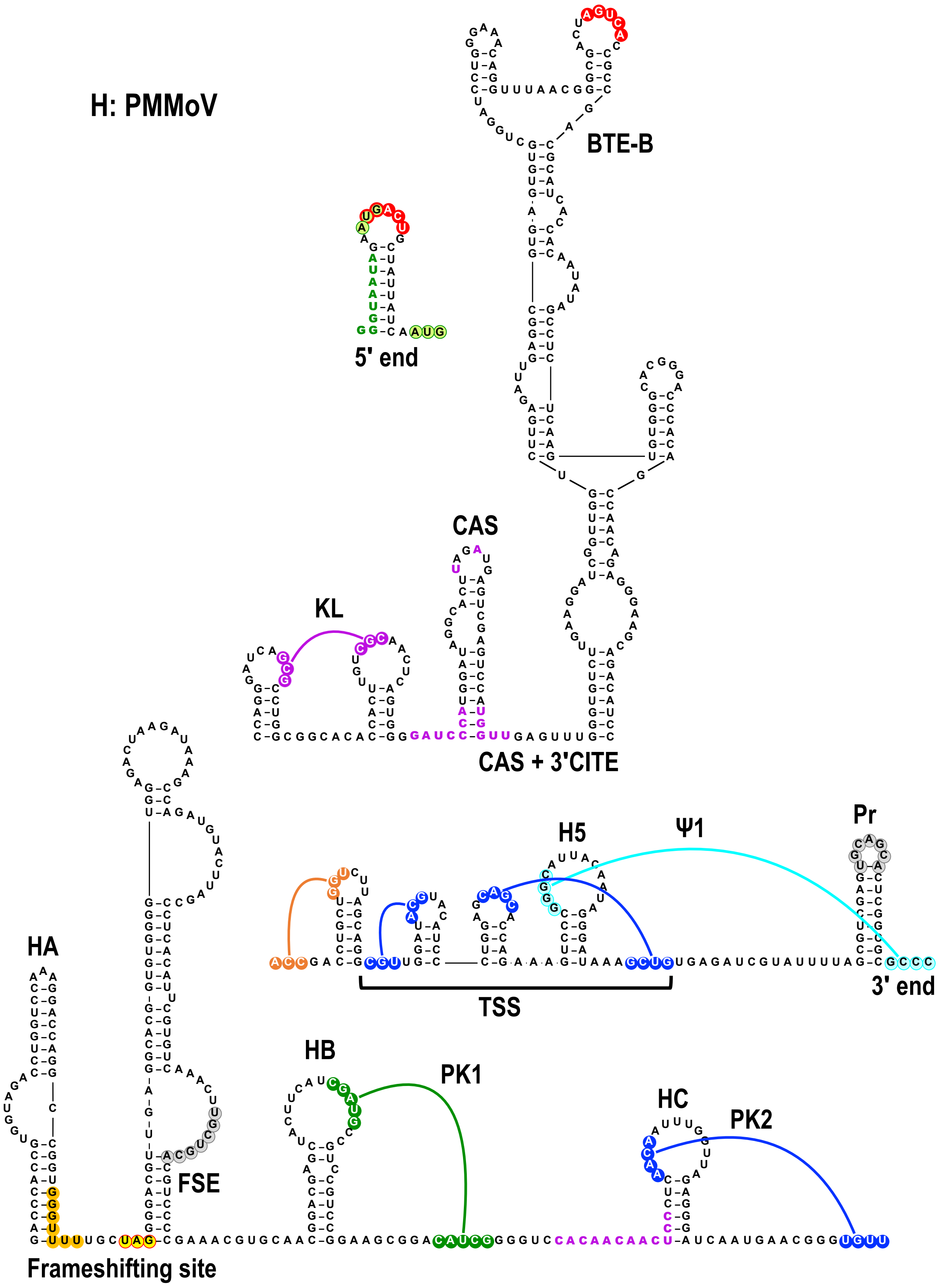

Supplemental Figure 4H

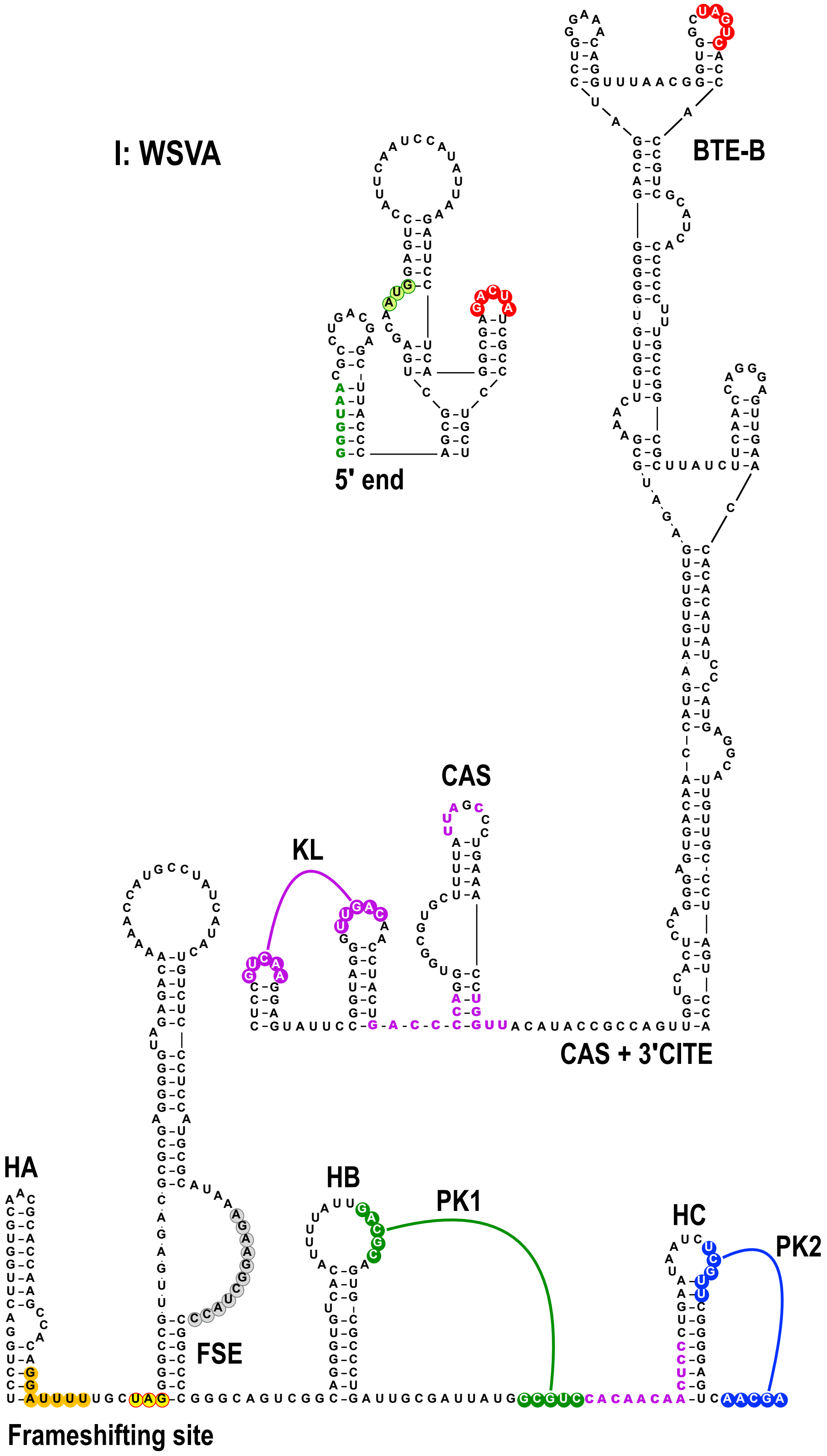

Supplemental Figure 4I

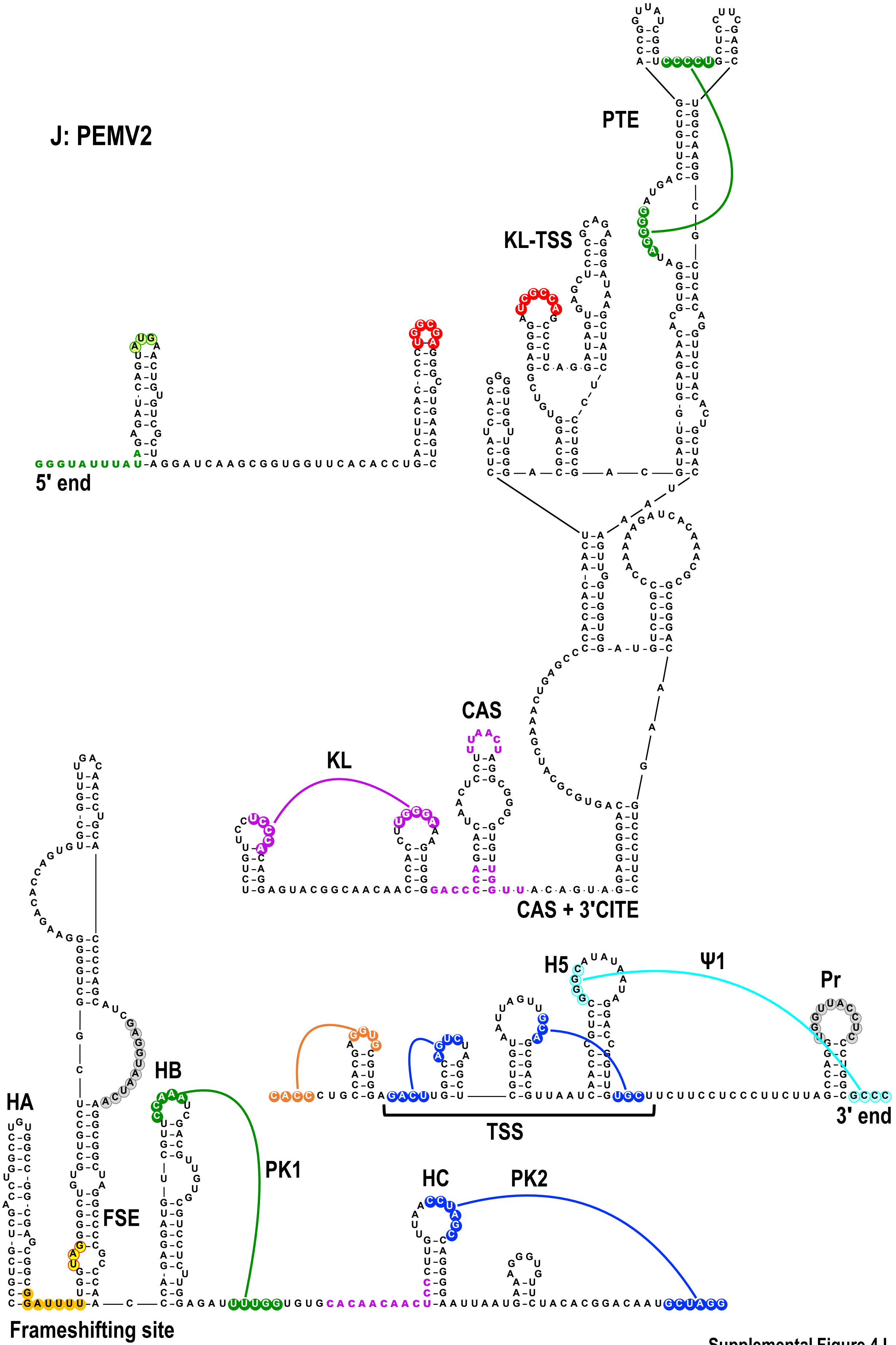

Supplemental Figure 4J

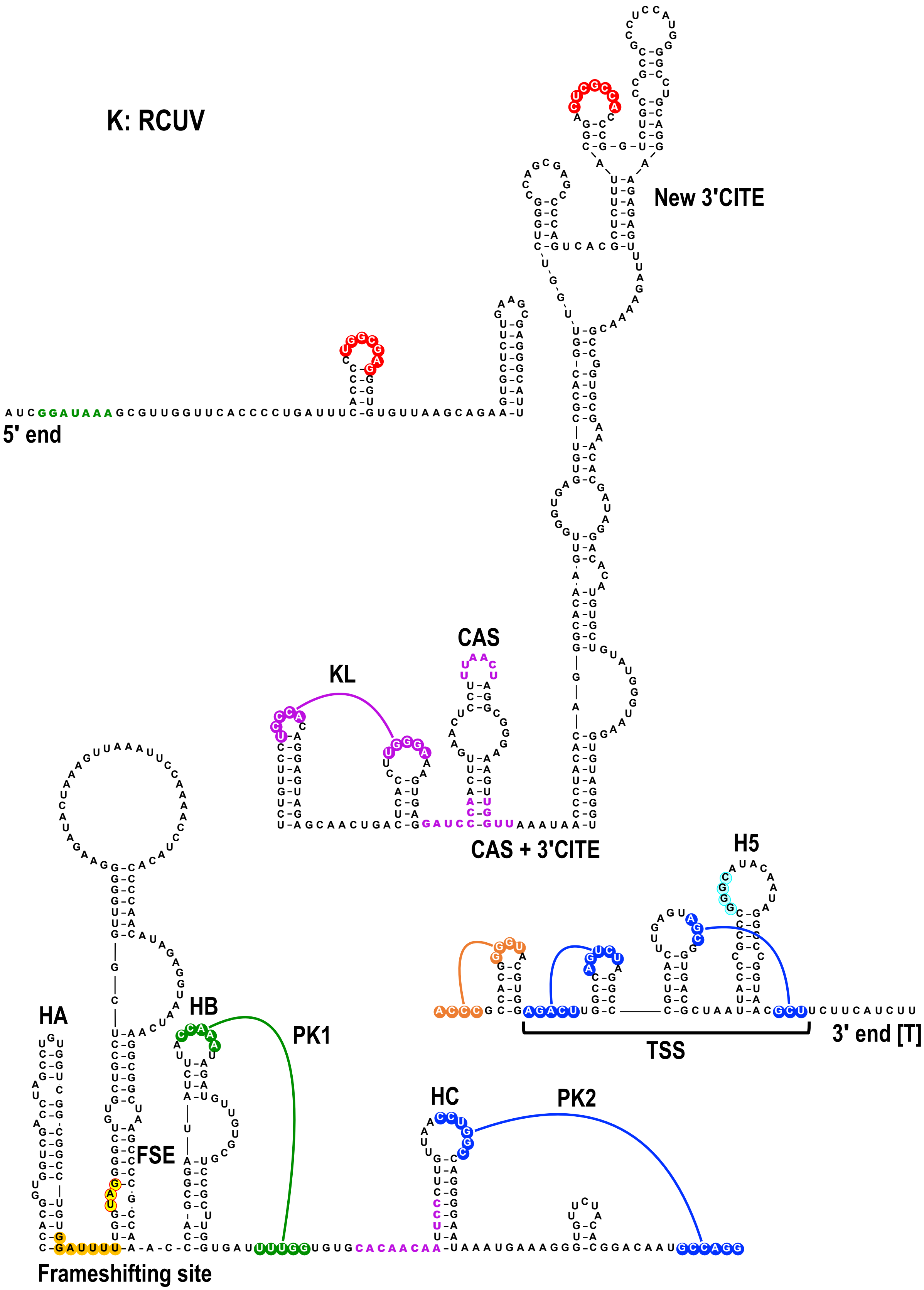

Supplemental Figure 4K

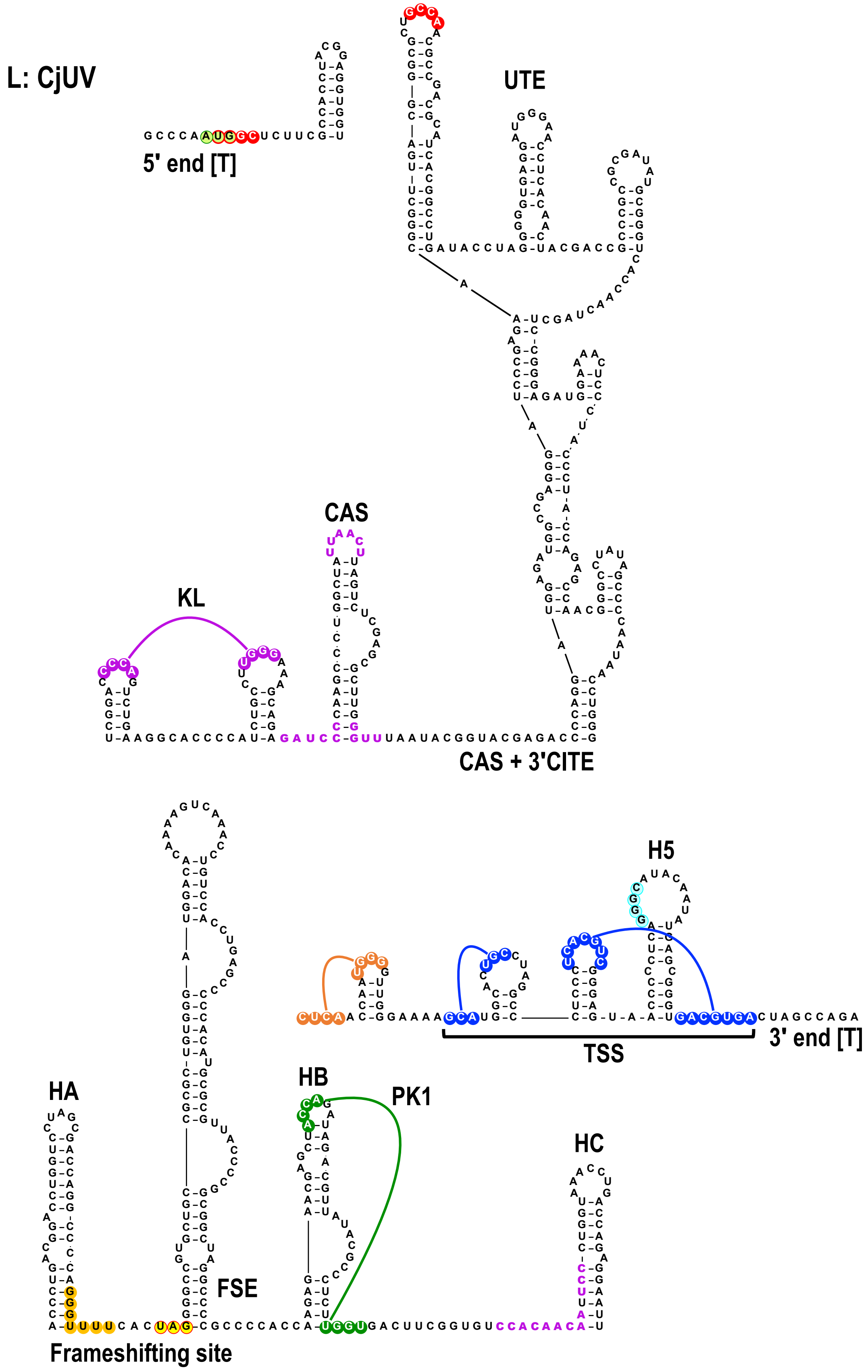

Supplemental Figure 4L

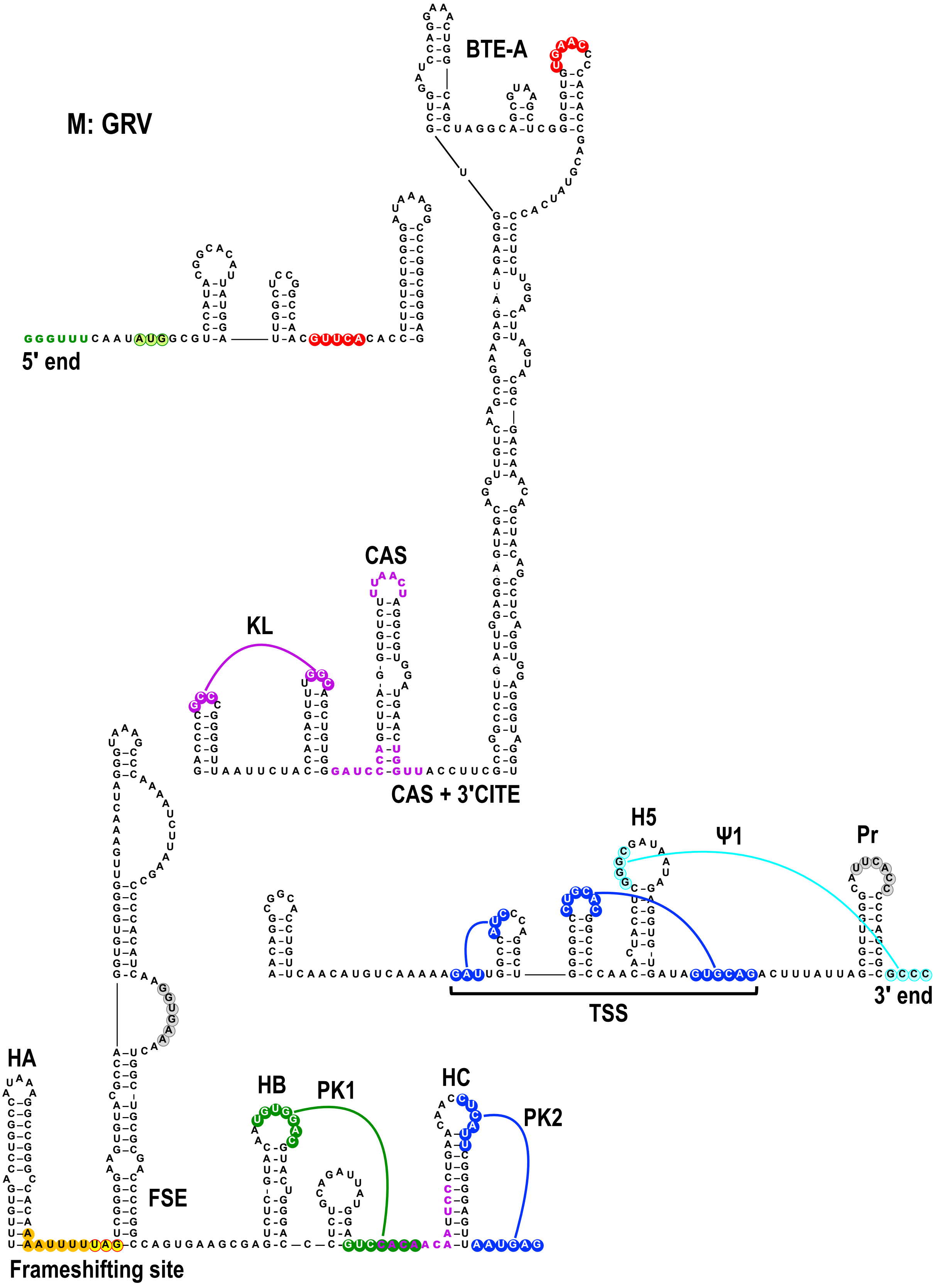

Supplemental Figure 4M

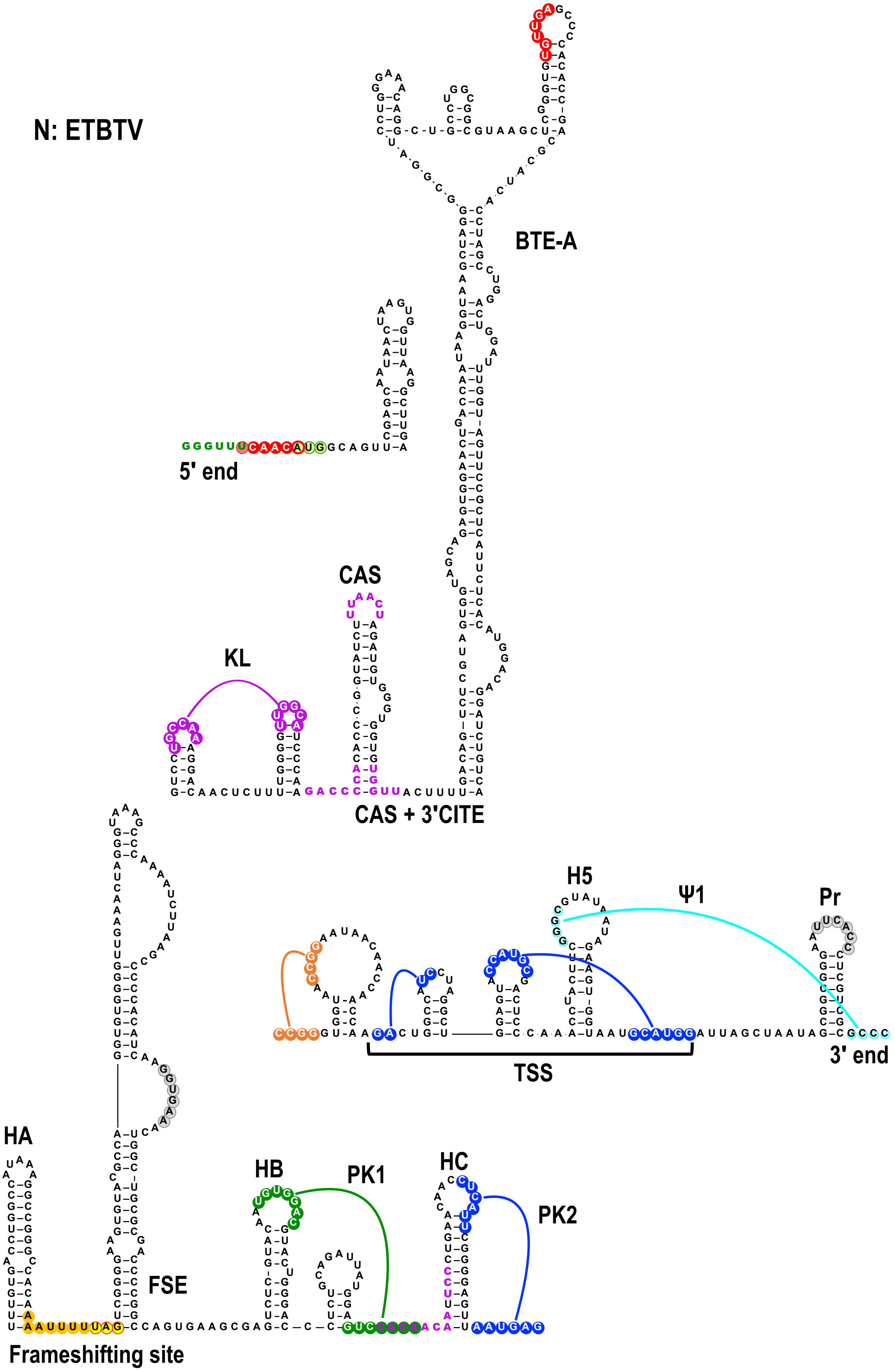

Supplemental Figure 4N

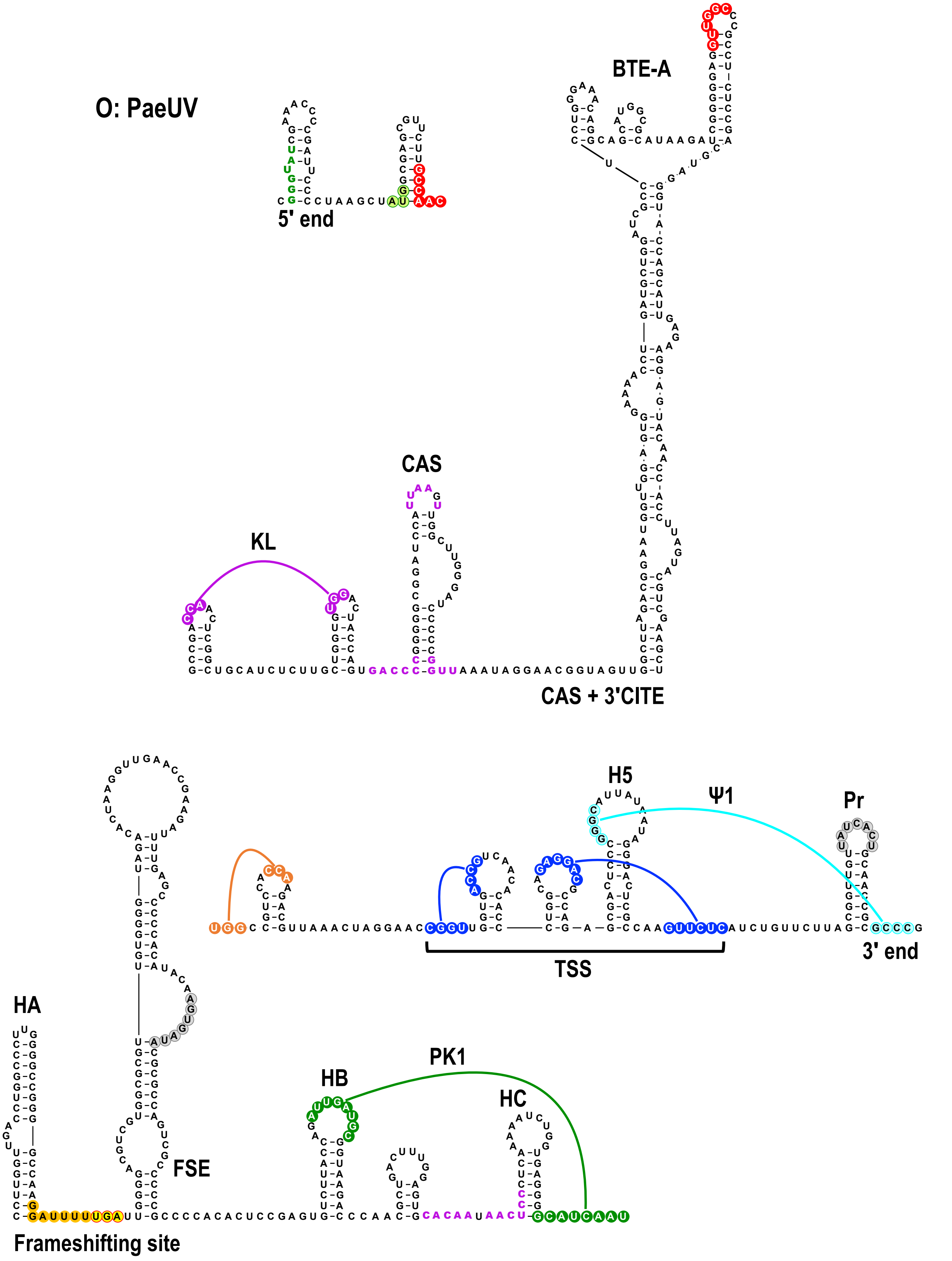

Supplemental Figure 4O

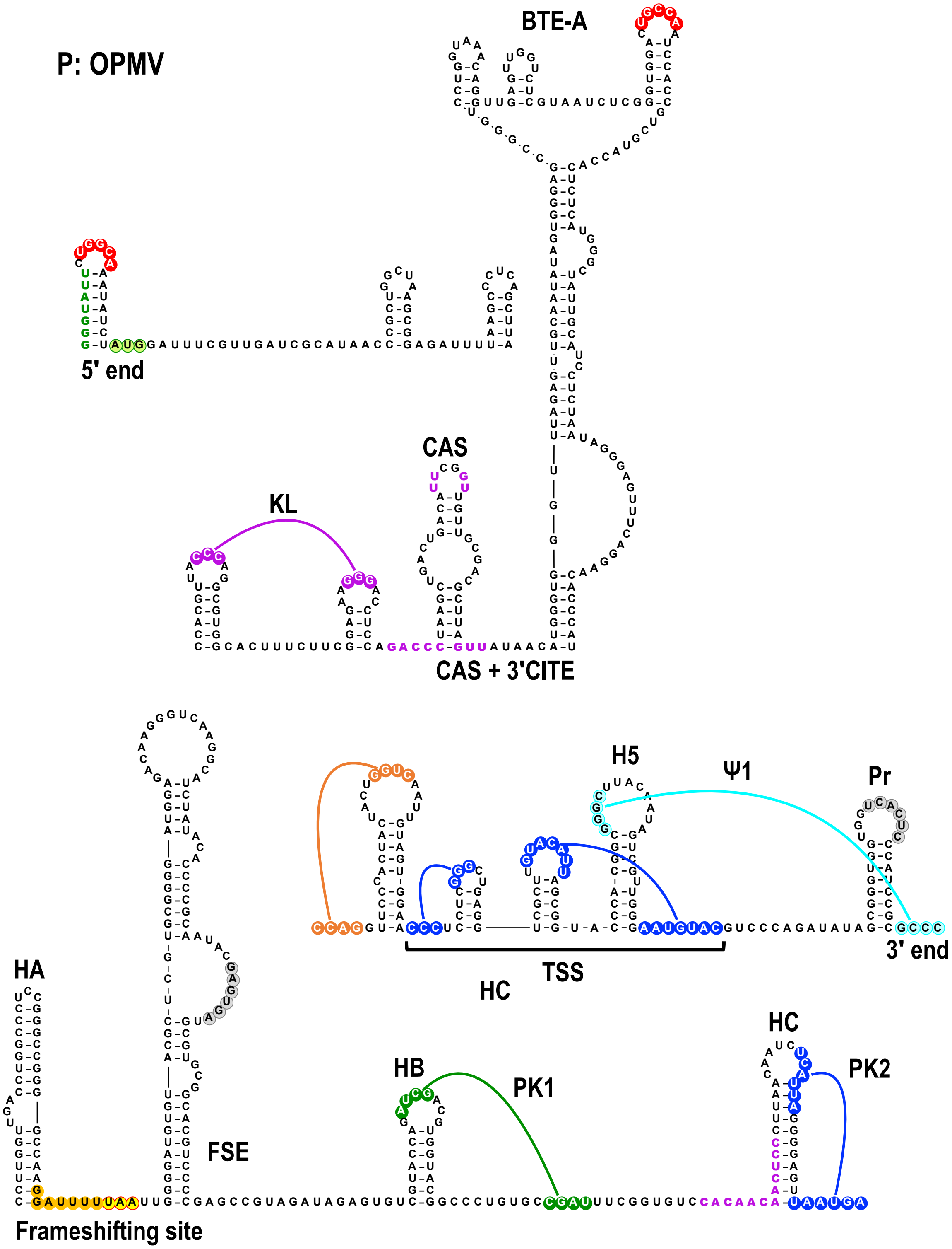

Supplemental Figure 4P

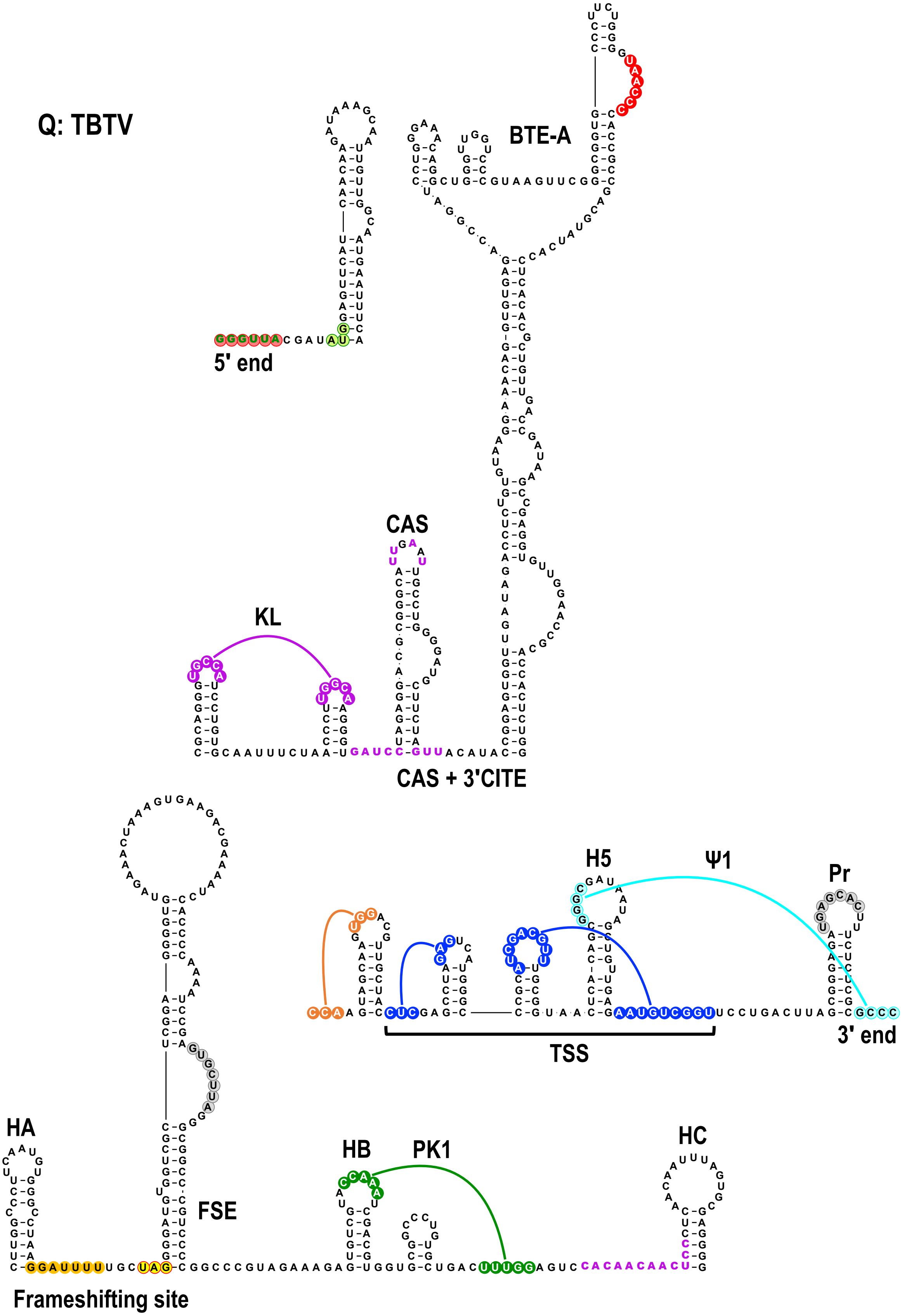

Supplemental Figure 4Q

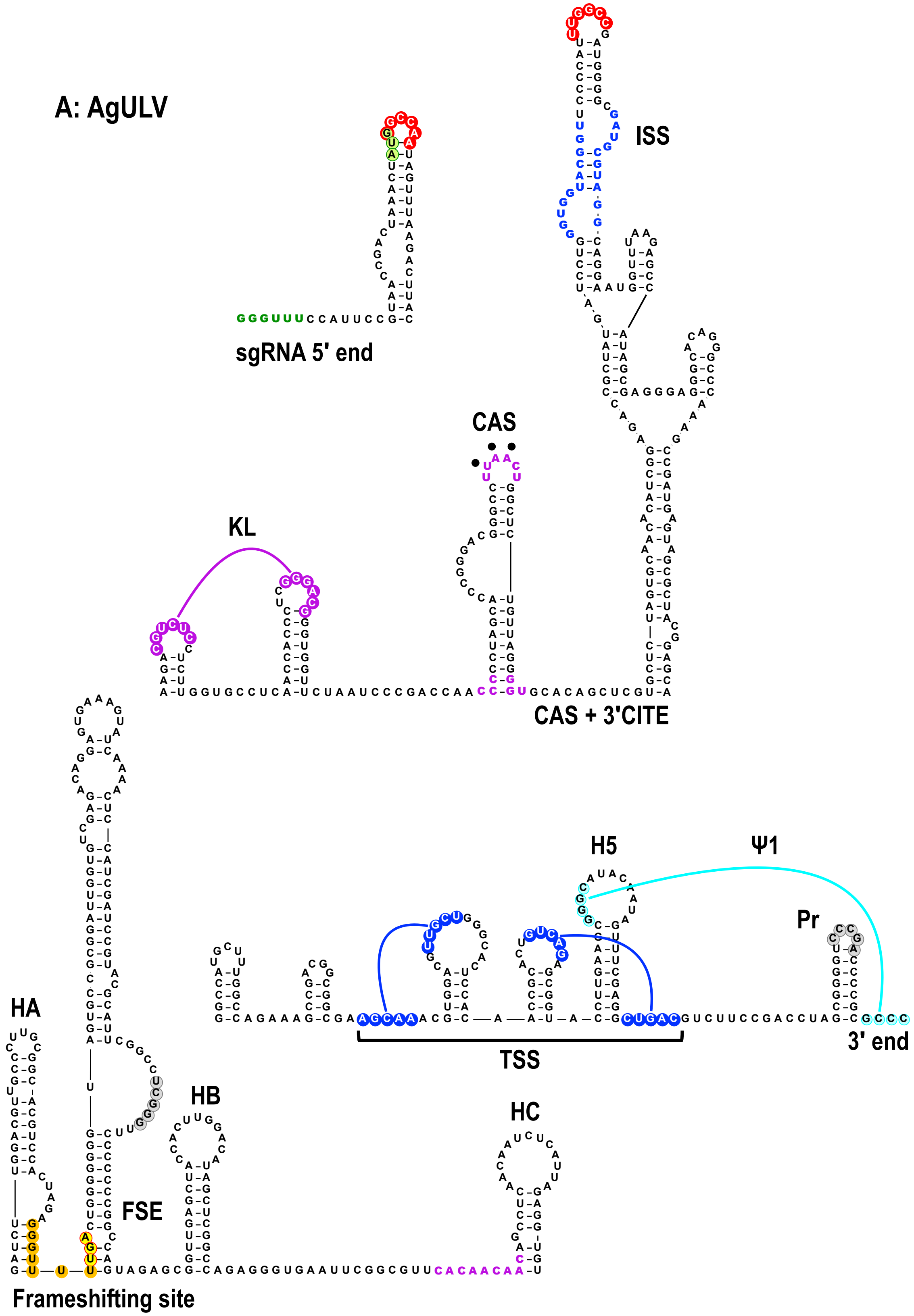

Supplemental Figure 5A

B: ArULV

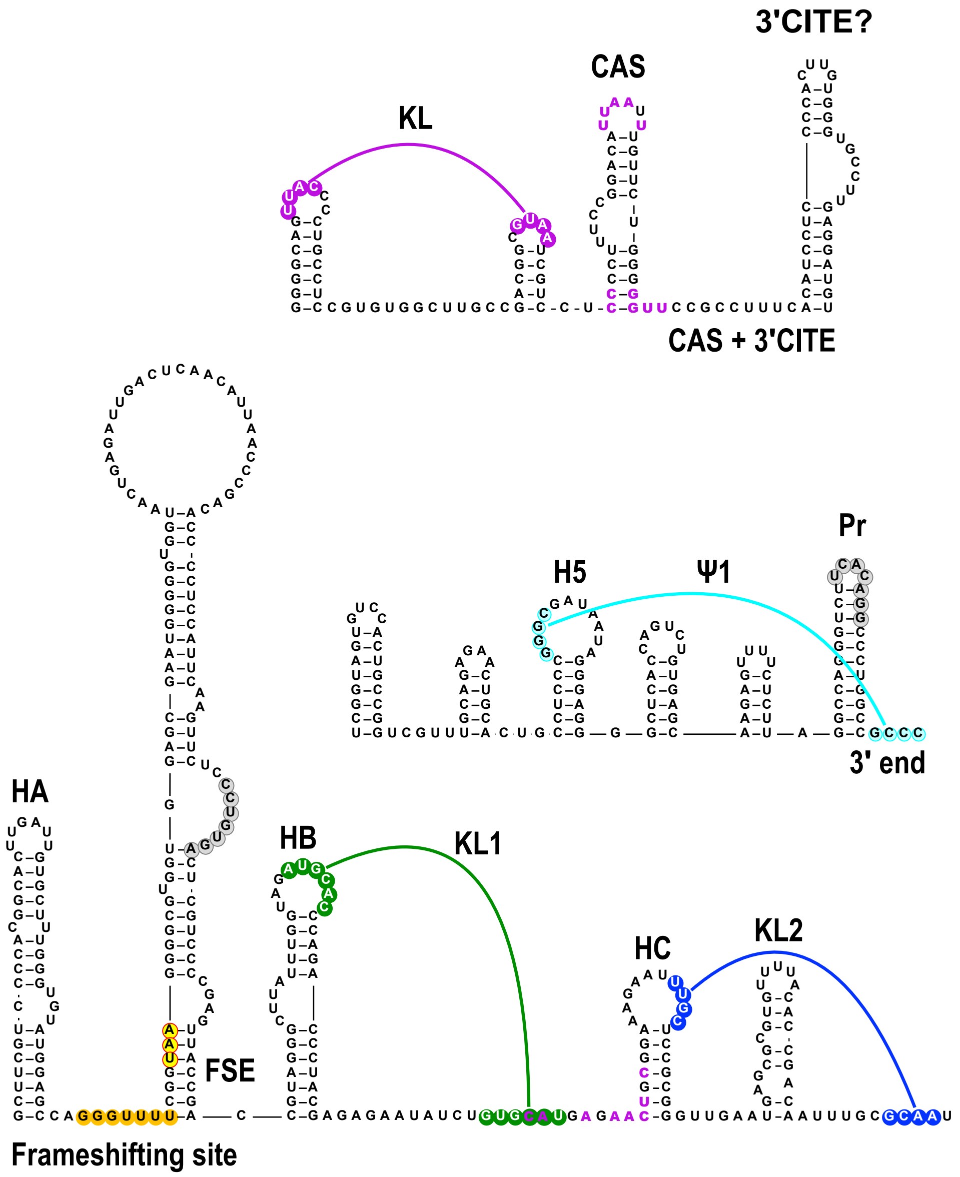
3'CITE?
CAS
KL
CAS + 3'CITE
Pr
H5
Ψ1
3' end
HA
HB
KL1
HC
KL2
FSE
Frameshifting site

C: PaULV1

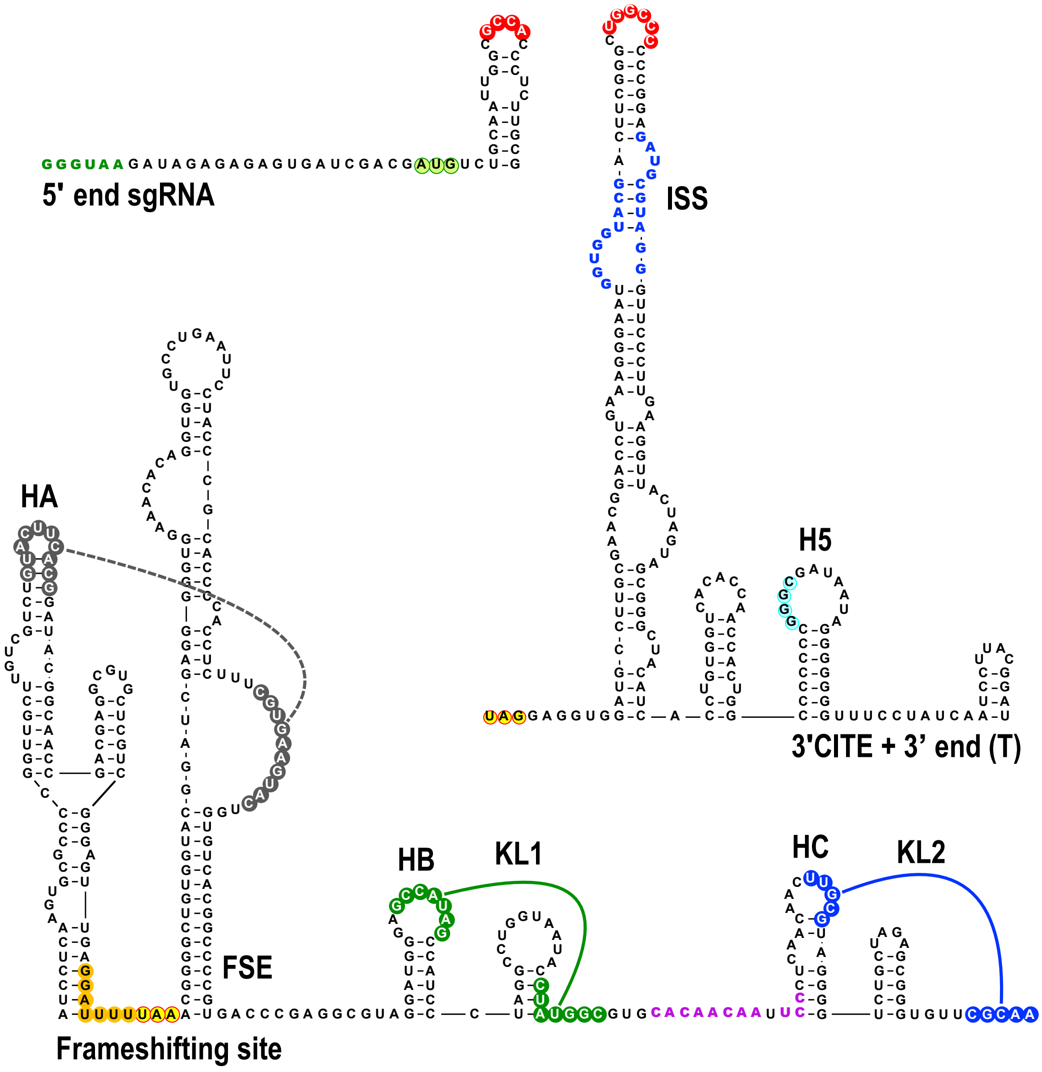

Supplemental Figure 5C

D: SgULV1

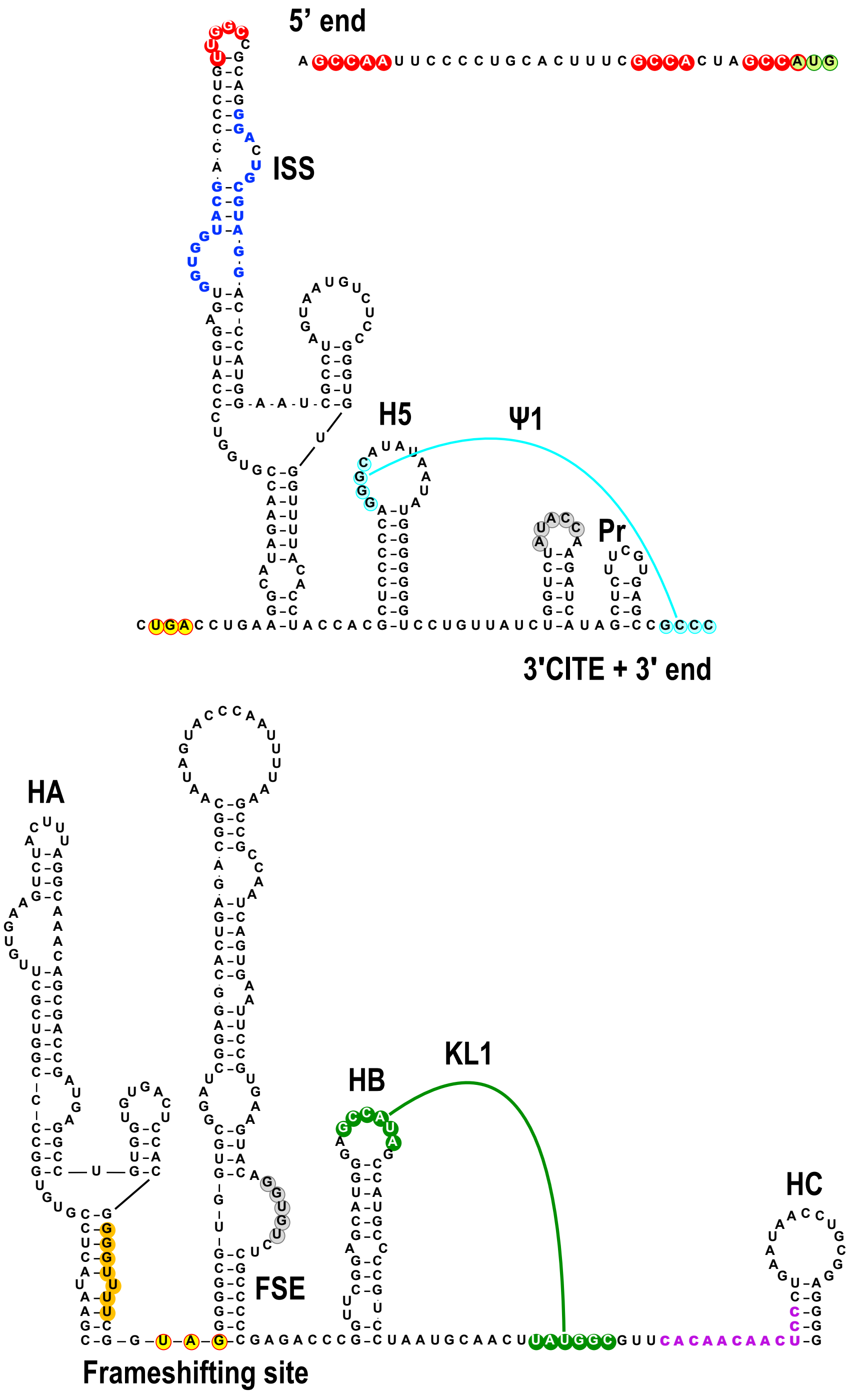

5’ end
AGCCAAUUCCCCUGCACUUUCGCCACUAGCCAUG
ISS
H5
Ψ1
Pr
3'CITE + 3' end
HA
HB
KL1
HC
FSE
Frameshifting site

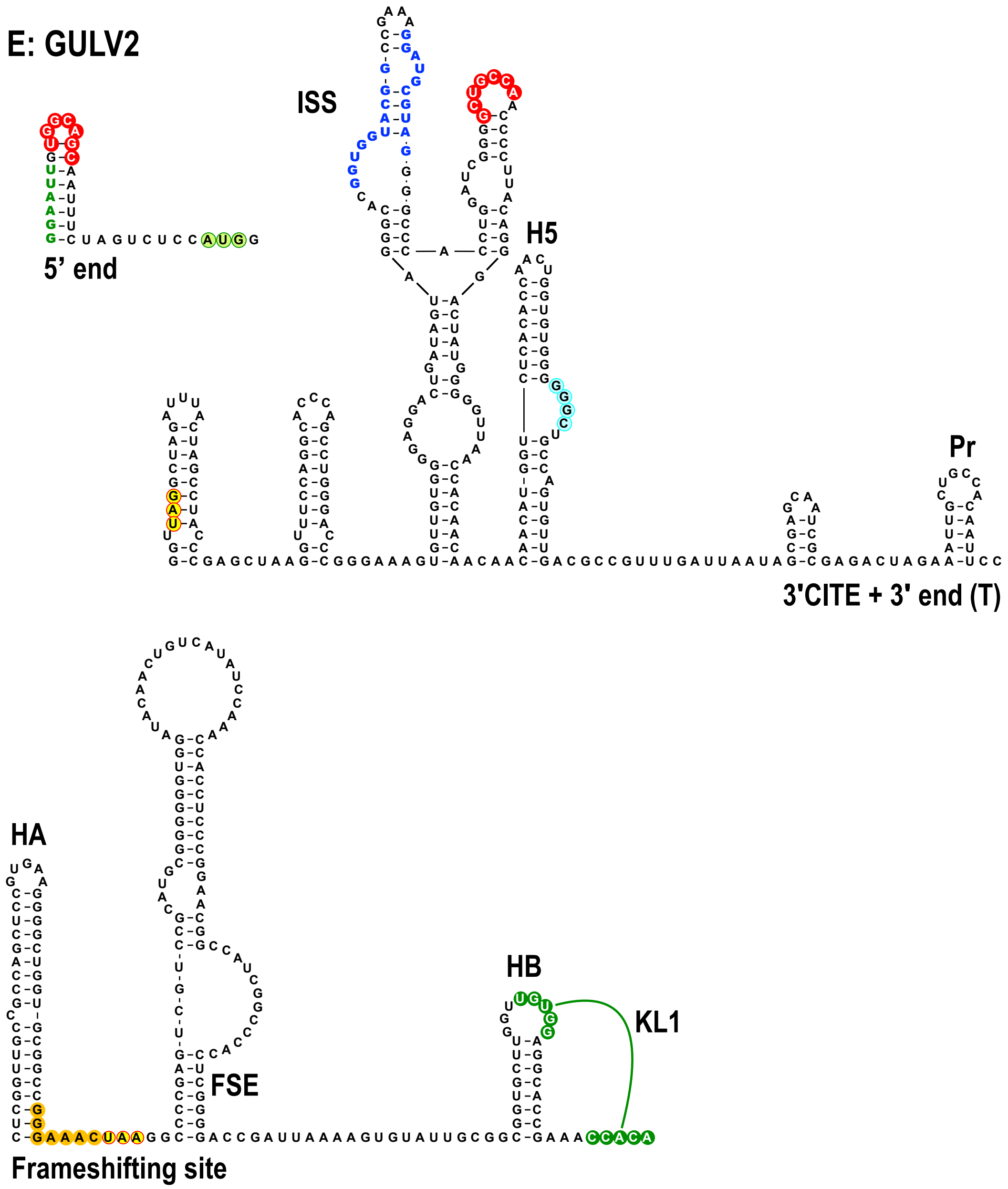

Supplemental Figure 5E

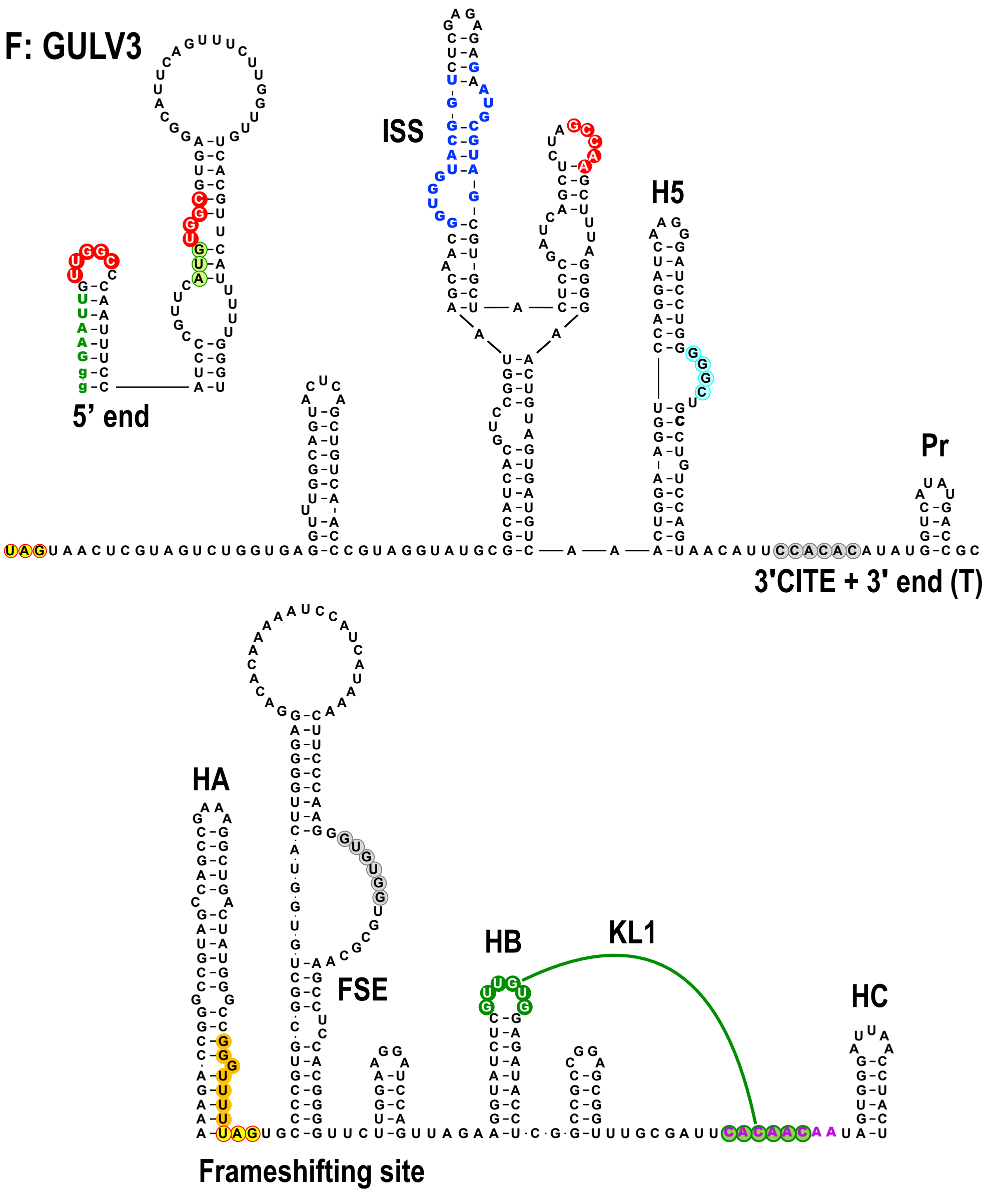

Supplemental Figure 5F

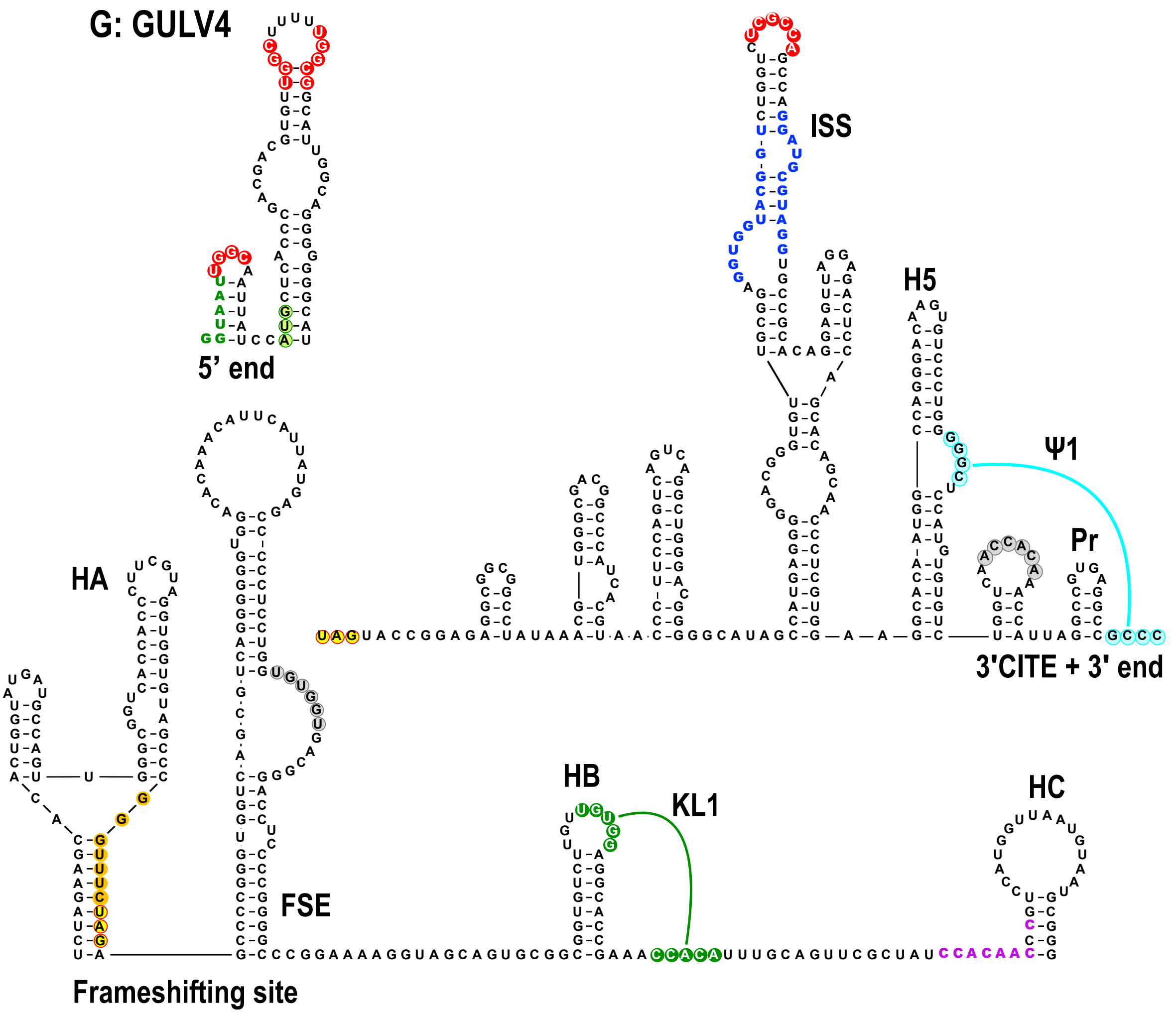

Supplemental Figure 5G

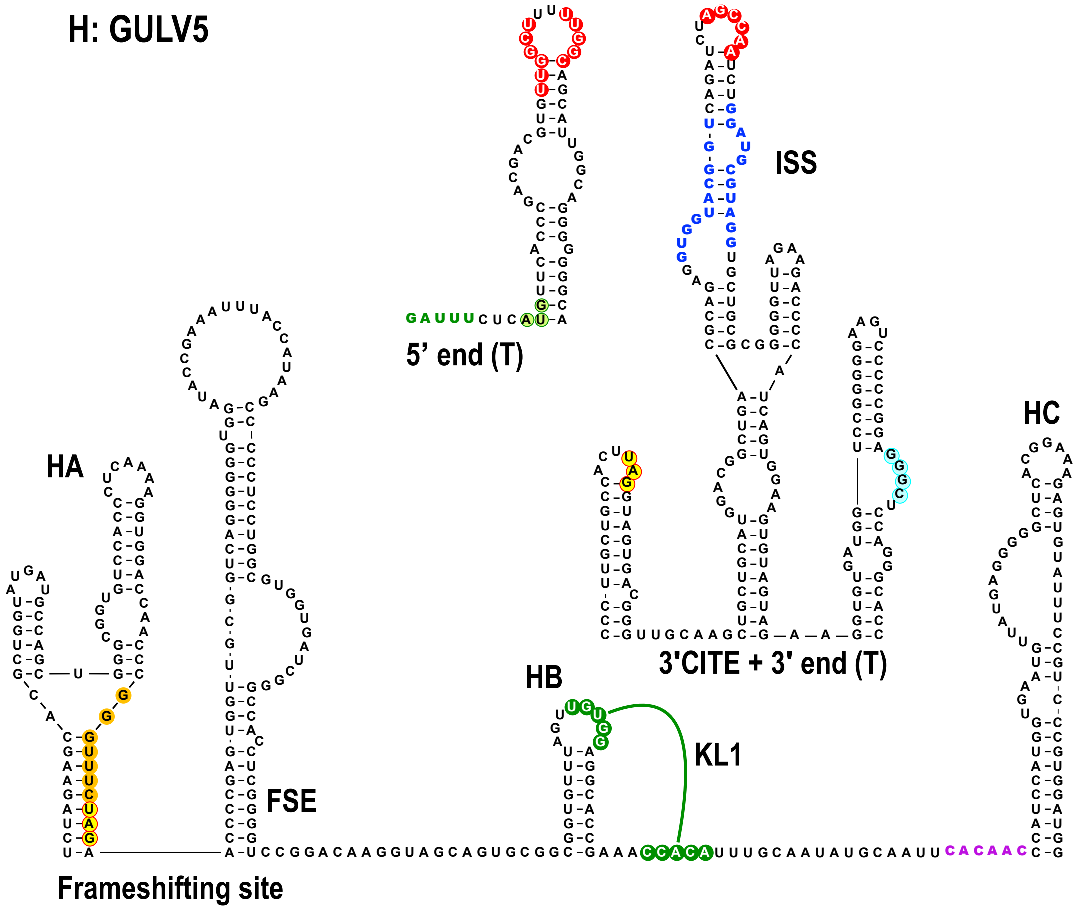

Supplemental Figure 5H

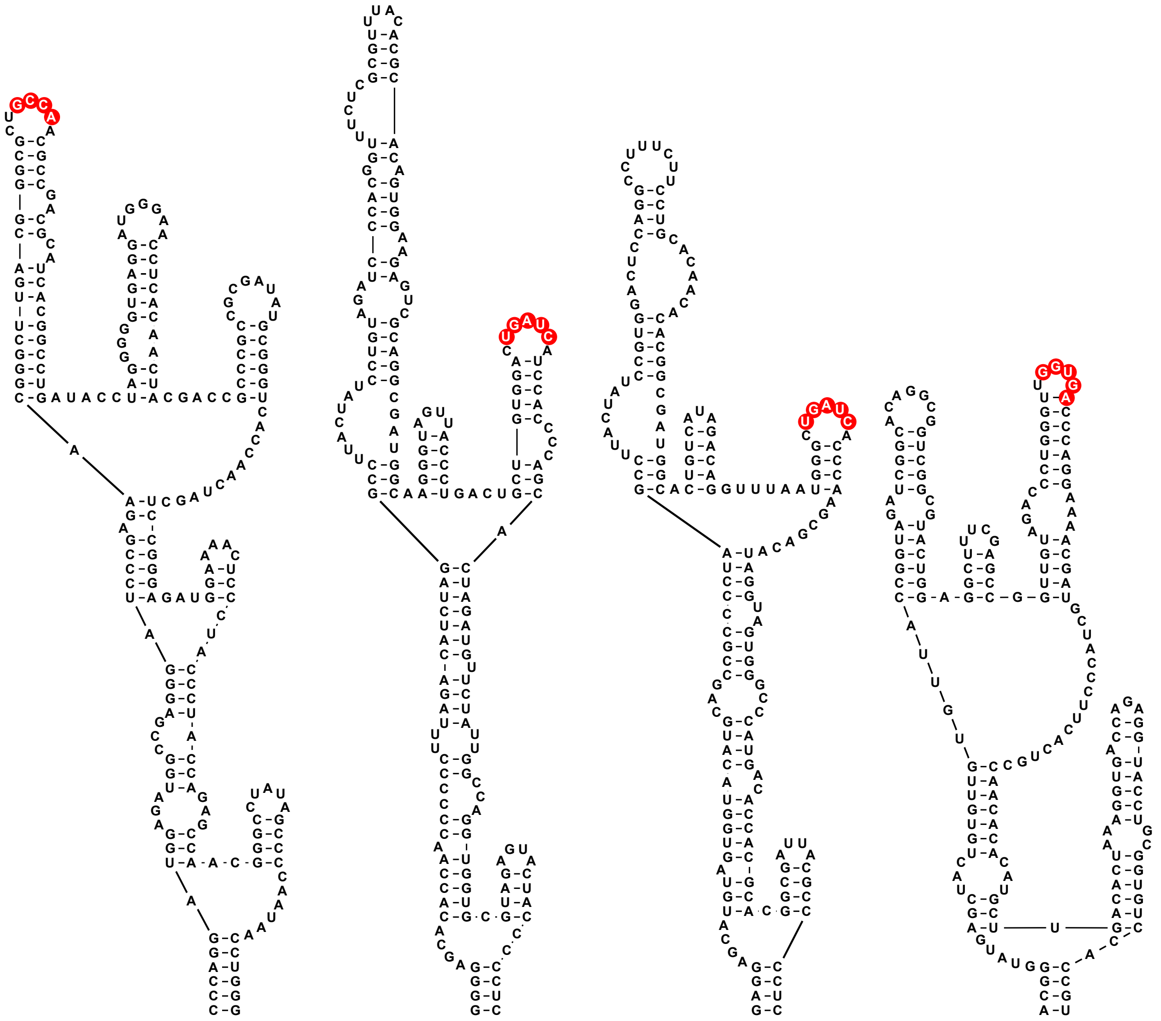

CJTLV PasUV CMoV CUV + WCMoV

Supplemental Figure 6
